# Pharmacological Characterization of GLPG3667, a Tyrosine Kinase 2-Selective Inhibitor, for the Treatment of Inflammatory and Autoimmune Diseases

**DOI:** 10.1007/s10753-026-02519-1

**Published:** 2026-05-16

**Authors:** Céline Cottereaux, May-Linda Lepage, Isabelle Parent, Maikel Colli, An Van de Water, Emilie Lagoutte, Christelle David, Maarten Van Balen, Adrien Cosson, Roland Blanque, Kenji Shoji, Mia Jans, Laetitia Furio, Steven Van der Plas, Reginald Brys, David Amantini, René Galien

**Affiliations:** 1 Galapagos SASU, 102 Avenue Gaston Roussel, Romainville, 93230 France; 2 Present Address: NovAliX, Romainville, France; 3 Galapagos NV, Generaal De Wittelaan L11 A3, Mechelen, 2800 Belgium; 4 Present Address: Calida Therapeutics, Paris, France; 5 Present Address: ISS AG, Integrated Scientific Services, Biel/Bienne, Switzerland; 6 Present Address: Oncodesign Precision Medicine, Dijon, France; 7 Present Address: iTeos Therapeutics, Gosselies, Belgium; 8 Present Address: Agomab Therapeutics, Gent, Belgium

**Keywords:** TYK2, Selective inhibitor, IFN-α challenge, Autoimmune diseases, JAK

## Abstract

Janus kinase inhibitors have demonstrated efficacy in treating inflammatory and autoimmune diseases; however, several concerns restrict their use in medical practice. One member of this kinase family, tyrosine kinase 2 (TYK2), recently attracted attention owing to the limited number of key inflammatory cytokine pathways it mediates (type I interferon, interleukin [IL]-12/IL-23, and IL-10 family pathways) and the numerous genetic studies associating polymorphisms of the *TYK2* gene with protection against several inflammatory and autoimmune diseases. In that regard, we discovered a selective, reversible, ATP-competitive inhibitor of TYK2, GLPG3667. We present the selectivity of GLPG3667 in biochemical, cellular, and human whole blood assays. In vitro, GLPG3667 inhibited the IL-12- and IL-23-induced polarization of T helper (T_H_)1 and T_H_17, respectively, but was shown to have a more limited impact on the IL-10 pathway and the anti-inflammatory effects of IL-10 in vitro. Pharmacodynamic analyses performed during phase 1 trials, in which healthy volunteers received GLPG3667 orally, confirmed the selectivity of the compound for TYK2-dependent pathways. In addition, following an in vivo challenge in these healthy volunteers, GLPG3667 inhibited the biological effects of interferon-α, supporting its potential use in treating several inflammatory and autoimmune diseases.

**Supplementary Information:**

The online version contains supplementary material available at 10.1007/s10753-026-02519-1.

## Introduction

Despite the increasing number and variety of treatments available, there is still a high level of unmet medical need for patients with inflammatory and autoimmune diseases. Many of these diseases do not have a cure, and patients generally must undergo lifelong treatments. Therefore, medications should have a good safety profile over a long period of time [1].

Therapeutic antibodies (biologics) are considered the first revolution in the treatment of inflammatory and autoimmune diseases, displaying high selectivity for their target and thus avoiding potential side effects due to off-target effects. This is combined with a long activity time, allowing for longer dosing intervals. However, the mode of administration (intravenous or subcutaneous injection) may be an issue for some patients, and in case of liability, washout periods are problematic [2].

Small molecules inhibiting Janus kinases (JAKs) were considered the second revolution in the treatment of inflammatory and autoimmune diseases. JAKs are nonreceptor tyrosine kinases that mediate the signaling of more than 60 cytokines and growth factors [3]. This family of proteins is composed of four members: JAK1, JAK2, JAK3, and tyrosine kinase 2 (TYK2). JAKs work through dimerization and phosphorylation of signal transducer and activator of transcription (STAT) factors, which are a family of seven proteins [4]. Mechanistically, the interaction of ligands with their cognate receptors leads to trans- and autophosphorylation of JAKs that are associated with the intracellular part of the receptors. Once activated, JAKs phosphorylate STAT factors, which dimerize, migrate to the cell nucleus, and activate the transcription of genes that are responsible for the effects of cytokines [5]. Blockade of the activity of one or several JAKs using a JAK inhibitor allows for alteration of the signaling of several cytokines, providing a broad anti-inflammatory effect, the spread of which depends on the selectivity of the molecule to inhibit one or more JAKs [6].

To date, more than 15 JAK inhibitors have been marketed for inflammatory or autoimmune diseases and display different selectivity features [7]. However, safety issues (mainly malignancies and cardiovascular diseases) limit their use for rheumatic and dermatologic conditions, and molecules without such concerns are forthcoming [8].

TYK2 is an interesting member of the JAK family because it is used by cytokines that have limited and well-characterized roles in inflammation and immunity. It mediates the signaling of type I interferons (IFNs), such as the IFN-α/β, as well as the cytokines interleukin (IL)-12 and IL-23, and the IL-10 family of cytokines, with various degrees of requirement for these signaling [9]. So far, studies of various selective TYK2 inhibitors have shown that they can fully inhibit type I IFN and IL-12/IL-23 signaling, whereas they are less effective in inhibiting the IL-10 family signaling pathway in some cases [10–12]. Interestingly, many genome-wide association studies have reported the association between several variants of TYK2 and inflammatory, autoimmune, or fibrotic diseases and cancer [13–19]. TYK2-mutant or -deficient mice have also provided evidence for the key role of this kinase in inflammatory diseases such as colitis, psoriasis, and arthritis [20–22]. In addition, clinical trials of the selective TYK2 allosteric inhibitor deucravacitinib demonstrated efficacy in targeting this kinase for the treatment of patients with psoriasis, psoriatic arthritis, and systemic lupus erythematosus (SLE) [23–25]. Ropsacitinib, another TYK2-selective inhibitor, demonstrated an acceptable safety profile and efficacy in patients with psoriasis [26]. More recently, the selective TYK2 inhibitor zasocitinib (TAK-279) also displayed efficacy in patients with psoriasis [27]. These data confirm the clinical relevance of the type I IFN and IL-12/IL-23 pathways and are in line with the efficacy of anifrolumab and ustekinumab. Anifrolumab is a monoclonal antibody which blocks IFN-α receptor subunit 1 and is approved for the treatment of patients with SLE [28]. Ustekinumab is a monoclonal antibody targeting IL-12, and IL-23 through IL-12p40, approved for the treatment of patients with psoriasis [29], psoriatic arthritis [30], and inflammatory bowel disease [31, 32]. Both anifrolumab and ustekinumab displayed acceptable safety profiles and efficacy in clinical trials, indicating that targeting TYK2 should provide efficient treatment, combined with a good safety profile.

Herein, we report the pharmacological characterization of GLPG3667, a reversible and selective TYK2 inhibitor, which is currently in development for the treatment of patients with dermatomyositis (DM) and SLE.

## Materials and Methods

### Ethics Statement

Clinical studies NCT04097938 and NCT04736927 were approved by the OLV Ziekenhuis Ethisch Comité (Belgium; approval number 2019/065) and the Wales Research Ethics Committee 2 (approval number 20/WA/0335), respectively. The studies complied with all relevant ethical regulations, and all participants provided written informed consent to participate in these studies.

### Biochemical Assays

#### ³³P (Phosphorus-33) Radioactive Filter Plate Assay for TYK2

Five microliters of a dilution series of compound, starting from the highest final concentration of 20 µM, 1/5 dilution, was added to the wells of a 96-well V-bottom plate. TYK2 (Carna Biosciences, Kobe, Japan) and polyGT (Eurogentec, Seraing, Belgium) were used at final concentrations of 240 ng/mL and 0.05 mg/mL, respectively. The enzyme and substrate were diluted in 25 mM MOPS pH 7.2, 50 mM NaCl, 0.01% Brij-35, 0.5 mM EDTA, 1 mM DTT, 5 mM MnCl_2_, and 10 mM MgCl_2_ in a total volume of 11 µL. The reactions were started by adding 9 µL ATP mixture, consisting of 0.1 µM unlabeled ATP and 0.125 µCi/25 µL [γ−33P]-ATP diluted in the same buffer, to the assay plate. Plates were incubated at 30 °C for 120 min. The reactions were stopped by adding 25 µL phosphoric acid (150 mM) to the reactions. The terminated kinase reactions were transferred using a harvester on pre-wetted unifilter-96 plates. These filter plates were washed six times with phosphoric acid (75 mM). Forty microliters of MicroScint-20 was added to each well. Readout was performed on a TopCount instrument (PerkinElmer, Waltham, MA, USA).

#### ^33^P radioactive Filter Plate Assays for JAK3 (Galapagos), JAK3 (Eurofins)

Five microliters of a dilution series of compound, starting from the highest final concentration of 20 µM, 1/5 dilution, was added to the wells of a 96-well V-bottom plate. JAK3 (Carna Biosciences) and polyGT (Eurogentec) were used at final concentrations of 20 ng/mL and 0.1 mg/mL, respectively. The enzyme and substrate were diluted in 25 mM Tris pH 7.5, 0.01% Triton X-100, 0.5 mM EGTA, 2.5 mM DTT, 0.5 mM Na_3_VO_4_, 5 mM β-glycerol phosphate, and 10 mM MgCl_2_ in a total volume of 11 µL. The reactions were started by adding 9 µL ATP mixture, consisting of 1 µM unlabeled ATP and 0.125 µCi/25 µL [γ−33P]-ATP diluted in the same buffer, to the assay plate. Plates were incubated at 30 °C for 45 min. The reactions were stopped by adding 25 µL phosphoric acid (150 mM) to the reactions. The terminated kinase reactions were transferred using a harvester on pre-wetted unifilter-96 plates. These filter plates were washed six times with phosphoric acid (75 mM). Forty microliters of MicroScint-20 was added to each well. Readout was performed on a TopCount instrument (PerkinElmer).

The principle of the JAK3 radioactive assay run at Eurofins is comparable to the radioactive assay run in-house. The assay conditions were not optimized upon transfer of the compound testing from Galapagos to Eurofins. Assay conditions defined by Eurofins were used with two main differences: the GGEEEEYFELVKKKK peptide was used as a substrate instead of polyGT, and the Eurofins assay buffer was used.

#### ADP-Glo Kinase Assay for TYK2

One microliter of a dilution series of compound, starting from the highest final concentration of 20 µM, 1/5 dilution, was added to the wells of a white 384-well assay plate. Ten nanograms of TYK2 enzyme (Carna Biosciences) diluted in assay buffer (25 mM MOPS pH 7.2, 50 mM NaCl, 0.01% Brij-35, 0.5 mM EDTA, 10 mM MgCl_2_, 1 mM DTT) was added to the assay plate. The reaction was started by adding 2 µL ATP diluted in the same assay buffer at a concentration equal to ATP K_m_ on the assay plate. The mixture was incubated at room temperature for 120 min. The reactions were stopped, and the unconsumed ATP was depleted by adding 5 µL ADP-Glo reagent (Promega, Madison, WI, USA) to the reaction. The plates were incubated at room temperature for 40 min. ADP was converted to ATP, and luciferase and luciferin were introduced to detect ATP by adding 10 µL kinase detection reagent to the reaction. The plates were incubated at room temperature for 30 min. The luminescent signal was measured on an EnVision plate reader (PerkinElmer).

#### LANCE Time-Resolved Fluorescence Resonance Energy Transfer (FRET) Assays for JAK1, JAK2

Four microliters of a dilution series of compound, starting from the highest final concentration of 20 µM, 1/5 dilution, was added to the wells of a white 384-well assay plate. For the JAK1 assay, JAK1 (Invitrogen, Waltham, MA, USA) and LANCE Ultra ULight JAK1 (Tyr1023) Peptide (PerkinElmer) were used at final concentrations of 100 ng/mL and 20 nM, respectively. The enzyme and substrate were diluted in 15 mM MOPS pH 6.8, 0.01% Brij-35, 5 mM MgCl_2_, and 2 mM DTT in a total volume of 8 µL. For the JAK2 assay, JAK2 (Invitrogen) and LANCE Ultra ULight JAK1 (Tyr1023) Peptide (PerkinElmer) were used at final concentrations of 80 ng/mL and 25 nM, respectively. The enzyme and substrate were diluted in 25 mM MOPS pH 7.0, 0.01% Triton X-100, 7.5 mM MgCl_2_, and 2 mM DTT in a total volume of 8 µL. The reaction was started by the addition of 8 µL of 20 µM ATP for JAK1 and 0.3 µM ATP for JAK2 (final concentration, diluted in the same buffer as described above), and the mixture was incubated at room temperature for 60 min. The reaction was terminated by the addition of 20 µL detection mixture containing 10 mM EDTA and 0.5 nM LANCE Eu-W1024 anti-phosphotyrosine (PT66) (PerkinElmer) in 1x LANCE detection buffer. After 60 min of incubation at room temperature, light emission was measured on an EnVision plate reader (PerkinElmer).

### Cellular Assays

In all cell and whole blood assays, cytokine concentrations were preliminarily determined by generating a dose–response curve for each assay and cell type of interest. The EC90 was then determined and applied in the subsequent assays. In addition, responses to each cytokine were also determined at multiple time points to identify and use the time point that produced the highest level of cytokine induction.

#### Assays of the IFN-α, IL-2, and GM-CSF Pathways in Human Freshly Isolated PBMCs

Peripheral blood mononuclear cells (PBMCs) were isolated from the buffy coats of healthy donors who provided informed consent (Croatian Institute of Transfusion Medicine), by density gradient centrifugation using Lymphoprep (Axis-Shield Poc As Lymphoprep Solution, Axis-Shield, Dundee, UK). Cells to be triggered by IL-2 or granulocyte-macrophage colony-stimulating factor (GM-CSF) were resuspended in 10 mL culture medium (RPMI 1640 medium supplemented with 10% heat-inactivated fetal bovine serum (FBS) and 100 U/mL penicillin–streptomycin). Cells triggered by IFN-α were first resuspended in ammonium chloride lysate buffer (1.5 M NH_4_Cl, 100 mM NaHCO_3_, 10 mM Na_2_EDTA) and centrifuged (200 *g*, 10 min, 25 °C), and then resuspended in 50 mL of the culture medium mentioned above. PBMCs were seeded in a 96-well cell culture plate, and a serial dilution of compound (0.3% DMSO final) was added to the wells. The cells were preincubated with the compound for 30 min at 37 °C and then with the final concentrations of 0.05 ng/mL GM-CSF, 100 ng/mL IL-2, or 5 ng/mL IFN-α for 30 min at 37 °C.

For IFN-α- and IL-2-triggered cells, supernatants (containing lymphocyte cells) were transferred to a V-bottom polypropylene plate, and cells were lysed with the PathScan lysis buffer supplemented with protease inhibitor (Cell Signaling Technology, Danvers, MA, USA).

For GM-CSF-triggered cells, the plate content was mixed before transferring to the V-bottom plate, and cell lysis buffer was added to the cell pellet. Therefore, all cellular fractions were used for detection in the GM-CSF-induced phosphorylated STAT5 (pSTAT5) assay.

pSTAT1 and pSTAT5 were evaluated in the samples using PathScan ELISA according to the instructions of the supplier (Cell Signaling Technology).

#### Assays of the IFN-α, IL-12, IL-23, and IL-10 Pathways in Human Fresh Activated PBMCs

PBMCs were isolated from the buffy coats of healthy donors (Etablissement français du sang, Rungis, France) using density gradient centrifugation with Ficoll (Ficoll Paque Plus, GE HealthCare, Chicago, IL, USA). Cells were transferred into a cell culture flask (Cell Culture Flask 175 cm^2^, Corning, Amsterdam, Netherlands) at a cell density of 1 million cells/mL and were stimulated by the addition of 100 ng/mL phorbol 12-myristate 13-acetate (PMA, P8139, Sigma, Saint-Quentin-Fallavier, France) and 0.5 µM ionomycin (I3909, Sigma) in culture media (RPMI 1640 medium, Gibco, Carlsbad, CA, USA; 10% heat-inactivated FBS, Gibco; 100 U/mL penicillin–streptomycin, Gibco). The incubation was carried out for 4 days at 37 °C, 5% CO_2_. Twenty-four hours later, dead cells were removed using a 100 µM cell strainer, and the culture continued for 3 more days. On day 4, cells were centrifuged for 5 min at 300 *g* at room temperature and left in starvation media (RPMI with antibiotics) overnight.

GLPG3667 was added to fresh activated human PBMCs for a preincubation period of 30 min and IFN-α (Universal Type I IFN-Alpha, PBL Assay Science, Piscataway, NJ, USA), IL-10 (recombinant human IL-10, R&D Systems, Bio-Techne, Minneapolis, MN, USA), IL-12 (recombinant human IL-12, R&D Systems, Bio-Techne), and IL-23 (recombinant human IL-23, R&D Systems, Bio-Techne) were then added to stimulate the JAK signaling pathways.

pSTAT1 was evaluated using flow cytometry. Briefly, 0.5 million cells were dispensed into a polypropylene 96-well V-bottom plate (Microplate, 96 wells, PP V-bottom, Greiner Bio-One, Kremsmünster, Austria) and were incubated with GLPG3667 at different concentrations for 30 min at 37 °C under gentle agitation. Cells were then triggered with IFN-α (1000 UI/mL, PBL Assay Science) or vehicle (Dulbecco’s phosphate-buffered saline [DPBS] + 0.1% bovine serum albumin) for 30 min at 37 °C under gentle agitation. At the end of the incubation period, cells were fixed for 10 min at 37 °C with 170 µL of prewarmed 1X Lyse/Fix Buffer (BD Phosflow Lyse/Fix Buffer 5X, 558049, BD Biosciences, San Diego, CA, USA). After incubation, tubes were centrifuged at 400 *g* for 5 min at room temperature and were washed with DPBS. Next, cells were resuspended in 300 µL of ice-cold 100% methanol to permeabilize the cells, centrifuged at 400 *g* for 5 min at 4 °C, and washed with DPBS. The cell pellet was then resuspended in DPBS (100 µL), and 5 µL of anti-pSTAT1 coupled to PE (PE Mouse Anti-Stat1 [pY701] Clone 4a – 562069, BD Biosciences, Le Pont de Claix, France) was added and incubated for 30 min at room temperature in the dark and washed once with DPBS. Finally, all conditions were analyzed on a FACSLyric cytometer (BD Biosciences) gating 10,000 events per sample.

The evaluation of pSTAT3 and pSTAT4 was carried out using the MSD kit (Phospho-Stat Panel Multi-Spot 96-well 4-spot plate, MSD, Rockville, MD, USA). For differentiated cell sub-populations, the MSD platform permits the assessment of multiple endpoints using a substantially lower number of cells compared with traditional FACS-based measurements, with exception to pSTAT1, which was measured using FACS analysis as it was not included in the MSD kit. Briefly, 0.2 million activated PBMCs were dispensed into a polypropylene 96-well V-bottom plate and were incubated with GLPG3667 at different concentrations for 30 min at 37 °C under gentle agitation. After this incubation, cells were triggered with IL-23 (30 ng/mL, recombinant human IL-23, R&D Systems), IL-12 (10 ng/mL, recombinant human IL-12, R&D Systems), or IL-10 (10 ng/mL, recombinant human IL-10, R&D Systems). Next, cells were centrifuged, and the supernatant was discarded. Then, 100 µL of cold lysis buffer provided in the kit was added to the samples, incubated for 15 min on ice, and centrifuged at 400 *g* for 10 min at 4 °C. Detection of STAT phosphorylation was performed on clarified lysates as recommended by the supplier, and the plate was read out in a QuickPlex SQ 120 reader (MSD). Flow cytometry data were analyzed with the FlowJo software and displayed as the percentage of pSTAT1-positive cells. Data were finally expressed as the percentage of inhibition, calculated within the Excel software, using the formula below.

$$\begin{array}{c}Percentage\;of\;inhibition=100\\\frac{(\%\;positive\;cells\;treatment\;condition\;\%\;positive\;cells\;without\;trigger)}{(\%\;positive\;cells\;with\;trigger\;alone\;\%\;positive\;cells\;\in\;cells\;without\;trigger)}\end{array}$$

For MSD samples, raw data quantified as a relative unit were directly obtained from an MSD multi-spot assay system for pSTAT3 and pSTAT4 and data were finally expressed as the percentage of inhibition.

Graphs and pIC_50_ calculations were performed with the Prism 8 software (GraphPad). Data were presented individually for each donor and finally as a mean of the individual pIC_50_ ± standard error of the mean (− log IC_50_ [M]) and the corresponding IC_50_ (nM).

#### Assay of the IL-22 Pathway in the HT-29 Cell Line

HT-29 cells (HTB-38, ATCC) were maintained in culture in complete RPMI medium supplemented with 10% FBS and 100 U/mL antibiotics until serum starvation overnight before the assay. After starvation, cells were dispensed in starvation media in a polypropylene 96-well V-bottom plate and were incubated with GLPG3667 at different concentrations for 30 min at 37 °C under gentle agitation. Cells were then triggered with 100 ng/mL IL-22 (recombinant human IL-22, 200 − 22, PeproTech, Neuilly-sur-Seine, France) for 30 min at 37 °C under gentle agitation. Cells were then fixed and permeabilized as described above, and STAT1 or STAT3 phosphorylation was detected with an anti-pSTAT1 antibody coupled to PE (PE Mouse Anti-Stat1 [pY701], BD Biosciences) or an anti-pSTAT3 antibody coupled to AF488 (Alexa Fluor 488 Mouse Anti-Stat3 [pY705], BD Biosciences), respectively. Fluorescence was evaluated using flow cytometry, and data were analyzed as described above.

#### Assay of the IL-4 and IL-13 Pathways in the NCI-H1650 Cell Line

The NCI-H1650 cell line (CRL-5883, ATCC) was maintained routinely in culture in complete RPMI medium supplemented with 10% FBS and 100 U/mL antibiotics until serum starvation overnight before the assay. GLPG3667 was tested at different doses and incubated 30 min before the addition of the trigger. To evaluate the role of TYK2 in the IL-4 and IL-13 pathways, cells were stimulated with 10 ng/mL IL-4 (recombinant human IL-4, R&D Systems) or 30 ng/mL IL-13 (recombinant human IL-13, R&D Systems) for 30 min under gentle agitation. The activity of TYK2 was evaluated by measuring STAT6 phosphorylation using an anti-pSTAT6 antibody coupled to PE (PE Mouse Anti-Stat6 [pY641], BD Biosciences, France). Data were analyzed and displayed as described above.

#### Effect of GLPG3667 on T_H_1 and T_H_17 Polarization

PBMCs were isolated from the buffy coats of healthy donors as described above. Untouched CD4^+^ T cells were further isolated by depletion of non-T helper (T_H_) and memory CD4^+^ T cells using a CD4^+^ T-cell isolation kit (Human CD4^+^ T Cell Isolation Kit, Miltenyi Biotec, Bergisch Gladbach, Germany) according to the supplier’s instructions. Isolated untouched CD4^+^ T cells were stimulated for 24 h in 96-well plates (Nunc MaxiSorp Immuno Assay 96-well plate, Roskilde, Denmark) coated with anti-CD3 (3 µg/mL of Purified NA/LE Mouse Anti-Human CD3, BD Pharmingen, San Diego, CA, USA) in the presence of cytokines and antibodies that drive differentiation into T_H_1 or T_H_17 subsets. To monitor the effects of the compound on T-cell differentiation, compounds were added at indicated concentrations at the start of T-cell differentiation along with T_H_1 and T_H_17 cocktails. Briefly, after selection and counting, cells were centrifuged at 400 *g* for 5 min at room temperature, and 0.4 million cells in RPMI medium supplemented with 10% FBS, 100 U/mL antibiotics, 2 mM GlutaMAX (GlutaMAX Supplement, Gibco), and 55 µM β-mercaptoethanol (2-mercaptoethanol, Gibco) were dispensed per well of a pre-coated plate and were incubated with GLPG3667 at different concentrations for 30 min at 37 °C.

For T_H_1 cell polarization, after the 30-min preincubation with the compound, cells were cultured for 4 days in the presence of 0.5 µg/mL anti-CD28 antibody (Purified NA/LE Mouse Anti-Human CD28, BD Pharmingen), 1 µg/mL anti-IL-4 antibody (human IL-4 antibody, R&D Systems), 5 ng/mL IL-2 (recombinant human IL-2, R&D Systems), and 10 ng/mL IL-12 (recombinant human IL-12 p70, PeproTech).

For T_H_17 cell polarization, after the 30-min preincubation with the compound, cells were cultured for 4 days in the presence of 10 µg/mL anti-CD28 antibodies (Purified NA/LE Mouse Anti-Human CD28, BD Pharmingen), 5 µg/mL anti-IL-4 antibody (human IL-4 antibody, R&D Systems), and 5 µg/mL anti-IFN-γ antibody (human IFN-γ antibody, R&D Systems). A mix of the following cytokines was also used: 30 ng/mL IL-6 (recombinant human IL-6, PeproTech), 20 ng/mL IL-1β (recombinant human IL-1β, PeproTech), 10 ng/mL TGF-β1 (recombinant human TGF-β1 HEK293-derived, PeproTech), and 30 ng/mL IL-23 (recombinant human IL-23, PeproTech).

After 96 h of incubation with the polarization medium, plates were centrifugated at 400 *g* for 5 min at room temperature. Supernatants were stored at − 80 °C until the evaluation of the analytes, and cell viability was checked using a cell viability assay (CellTiter-Glo Luminescent Cell Viability Assay, Promega, Madison, WI, USA), performed as recommended by the supplier. The impact of GLPG3667 on T-cell polarization induction was analyzed by measuring levels released in culture supernatant of IFN-γ (AlphaLISA Human IFN-γ Kit, AL208C, PerkinElmer–Revvity, Waltham, MA, USA) for the T_H_1 cocktail or IL-17A (AlphaLISA Human IL17A, AL219F, PerkinElmer–Revvity) for the T_H_17 cocktail. All plates were read using a luminescence plate reader (EnSight Multimode Microplate Reader, PerkinElmer–Revvity).

Once cell viability was validated, the IC_50_ was calculated accordingly for the different analytes using the method indicated above.

#### TNF-α Assay in Human Monocytes

PBMCs were isolated from buffy coats using SepMate Isolation Tubes (STEMCELL Technologies, Vancouver, Canada). Following red blood cell lysis, CD14^+^ monocytes were purified by positive selection (130-050-201, Miltenyi Biotec) and resuspended in DMEM F12 medium supplemented with 10% FBS, 100 U penicillin, and 100 µg/mL streptomycin. Three thousand monocytes were plated in a 384-well plate and incubated with increasing concentrations of GLPG3667 for 30 min. IL-10 (217-IL, R&D Systems) was added 1 h before triggering with 1 ng/mL lipopolysaccharide (LPS) (L2654, Sigma-Aldrich, St. Louis, MI, USA) for 15 h. When neutralizing anti-IL-10 antibody (AF-217-NA, R&D Systems) was used, it was incubated with IL-10 for 30 min before the mixture was added to the monocytes. Tumor necrosis factor-α (TNF-α) levels in the supernatant were determined using AlphaLISA (AL208F, PerkinElmer–Revvity).

### Human Whole Blood Assays

Written, informed consent was provided by healthy volunteers as described below, followed by whole blood collection by venipuncture into lithium heparin tubes; the tubes were then gently inverted several times to prevent clotting and were stabilized by incubating the tubes for 30 min at 37 °C under gentle agitation.

#### IFN-α-induced IP-10

Blood aliquots were dispensed into 2 mL deep well plates and incubated for 30 min at 37 °C with GLPG3667 compound tested at eight consecutive threefold dilutions starting from 30 µM. After the incubation period, release of IFN-γ-induced protein 10 (IP-10) was triggered with 3000 U/mL IFN-α (Universal Type I IFN-Alpha, 11100-1, PBL Assay Science, PBL) for 24 h at 37 °C. At the end of the incubation period, plates were centrifuged at 4 °C and plasma was collected. Release of IP-10 was measured using a homemade ELISA (capture antibody, MAB266, R&D Systems; detection antibody, BAF-266, R&D Systems; standard recombinant human IP-10 protein, 266-IP, R&D Systems).

#### IL-12 + IL-18-induced IFN-γ Release

Blood aliquots were dispensed into 2 mL deep well plates and incubated for 30 min at 37 °C with GLPG3667 compound tested at eight consecutive threefold dilutions starting from 30 µM. After the incubation period, blood was triggered with 2 ng/mL IL-12 (recombinant human IL-12, 219-IL, R&D Systems) and 50 ng/mL IL-18 (recombinant human IL-18, 318-IL, R&D Systems) for 24 h at 37 °C. IFN-γ release was measured in plasma using ELISA (DuoSet Human IFN-gamma DY285B, R&D Systems).

#### IL-23 + IL-2 + IL18-induced IFN-γ Release

Blood aliquots were dispensed into 2 mL deep well plates and incubated for 30 min at 37 °C with GLPG3667 compound tested at 10 consecutive threefold dilutions starting from 30 µM. After the incubation period, blood was triggered with 10 ng/mL IL-2 (recombinant human IL-2, 202-IL, R&D Systems), 20 ng/mL IL-18 (recombinant human IL-18, 9124-IL, R&D Systems), and 0.5 ng/mL IL-23 (recombinant human IL-23, 1290-IL, R&D Systems) for 24 h at 37 °C. IFN-γ release was measured in plasma using ELISA (DuoSet Human IFN-γ, DY285B, R&D Systems).

#### Cytokine-induced pSTAT Measurement in Human Whole Blood

The procedure was the same as described by van Rompaey et al. [33]. Blood was preincubated for 30 min at 37 °C with GLPG3667 tested at 10 consecutive threefold dilutions starting from 30 µM, and then triggered with either 4 ng/mL IL-2 (recombinant human IL-2, 202-IL, R&D Systems), 3 ng/mL IL-6 (recombinant human IL-6, 206-IL, R&D Systems), 1000 IU/mL IFN-α (Universal Type I IFN-Alpha 2a, 11100-1, PBL Assay Science), 7 ng/mL IL-10 (recombinant human IL-10, 200 − 10, PeproTech), or 20 pg/mL GM-CSF (recombinant human GM-CSF, 7954-GM, R&D Systems), or vehicle (phosphate buffer saline + 0.1% bovine serum albumin) for 20 min at 37 °C under gentle agitation for IL-6, IL-2, GM-CSF, and IFN-α and 30 min at 37 °C under gentle agitation for IL-10. The anti-pSTAT and anti-CD antibodies used are listed in Supplementary Table 1.

### Pharmacodynamic Effects of GLPG3667 in Healthy Volunteers in Phase 1 Trials

#### Clinical Trials

The clinical study “A first-in-human randomized, double-blind, placebo-controlled study to evaluate the safety, tolerability, and pharmacokinetics of single and multiple ascending oral doses of GLPG3667 in adult, healthy, male subjects” (NCT04097938) was approved by the OLV Ziekenhuis Ethisch Comité (Belgium; approval number 2019/065). The study complied with all relevant ethical regulations, and all participants provided written informed consent. The compound and the placebo were administered orally once a day as a suspension (6 healthy volunteers received placebo and 6 received GLPG3667). pSTAT analysis was performed during the multiple ascending dose part of the study after blood collection at several time points, including pre-dose (day 1) and after a single administration of compound (day 1) or at steady state (day 10; Supplementary Fig. 1A). On day 11, an in vivo IFN-α challenge was performed, and blood was collected before and after GLPG3667 administration during the 5 following days.

The clinical study “An open-label, fixed-sequence, drug–drug interaction study in healthy subjects to evaluate the effect of GLPG3667 on the pharmacokinetics of midazolam, a sensitive index substrate of CYP3A4” (NCT04736927) was approved by the Wales Research Ethics Committee 2 (approval number 20/WA/0335). The study complied with all relevant ethical regulations, and all participants provided written informed consent. The compound was administered orally once a day as a tablet to 14 healthy volunteers. pSTAT analysis was performed after collection of blood pre-dose (day 1) and at steady state after 4 days of compound administration (day 6; Supplementary Fig. 1B).

Sampling was performed at steady-state during the two studies. In the first-in-human study, PK and PD samples were collected near the end of the dosing period to ensure that steady state had been achieved. GLPG3667 showed linear, time-independent pharmacokinetics across the evaluated dose range, with a half-life of approximately 9–15 h. Steady state was achieved after ~ 3 days of dosing, confirmed by inspection of trough levels. The drug–drug interaction study collected PK and PD samples after 4 days of dosing when steady-state had already been reached.

In both studies, GLPG3667 was well tolerated with no deaths or other serious adverse events; the majority of treatment emergent adverse events were mild to moderate and considered unrelated or likely unrelated to treatment.

#### pSTAT Analysis in Healthy Volunteers

Blood from participants receiving placebo or GLPG3667 was collected, triggered with cytokines (without addition of GLPG3667), and prepared for flow cytometry as described in the ‘Human Whole Blood Assays’ section. Flow cytometry data were analyzed with FACSDiva software and displayed as a percentage of pSTAT-positive cells among CD-positive cells. Briefly, a histogram plot for pSTAT1, pSTAT3, and pSTAT5 was created, and a sub-plot was gated on a stimulated cell population of interest. An unstimulated, pre-dose sample from each donor was used for setting the P3 gate for each trigger, and it contained fewer than 1% of positive events. Data were expressed as a percentage of pSTAT-positive cells among CD-positive cells and plotted using Excel software.

#### In vivo IFN-α Challenge in Healthy Volunteers

On day 11 of the multiple ascending dose part of the study in healthy volunteers, participants received a single subcutaneous injection of 1 million IU of IFN-α 2A (Intron A, Schering-Plough, Kenilworth, NJ, USA) in the abdomen in the 30 min after administration of GLPG3667 or placebo. The compound was administered again on day 12 and day 13, and blood was collected for serum preparation and in PAXgene tubes for gene expression analysis pre-dose on day 11 and at various time points up to 5 days after IFN-α injection (Supplementary Fig. 2).

#### Serum Neopterin Assay

After collection, serum tubes were incubated in an upright position at room temperature for 30–45 min to allow clotting. Serum was prepared by centrifugation and stored between − 36 °C and − 20 °C, for a maximum of 6 months, until analysis. Sample analysis was performed at ABL Lyon using Neopterin ELISA (IBL International, Hamburg, Germany) according to the technical manual of the manufacturer. Optical density values generated by the Infinite 200 plate reader were analyzed with Magellan software, and a 4-parameter logistic linear regression was used to analyze the calibration curve.

#### Transcriptomic Analysis of an in vivo IFN-α Challenge

Blood was collected by venipuncture directly in PAXgene Blood RNA Tubes (PreAnalytiX, Hombrechtikon, Switzerland) following the manufacturer’s instructions. Blood samples were further treated with a globin depletion kit to remove globin transcripts. Total RNA was obtained using a RNeasy kit following the manufacturer’s instructions (Qiagen, Venlo, Netherlands). Illumina sequencing libraries were prepared with polyA capture (mRNA + long non-coding RNA). After multiplexing the samples for each tissue (pooling multiple samples into a single sequencing run to maximize throughput, enable parallel processing, and potentially reduce batch effects as samples share conditions), the libraries were processed on an Illumina NovaSeq 6000 sequencer. Reads were mapped to the human reference transcriptome (hg38 build v100, Ensembl version; primary assembly) using the pseudoalignment model with Kallisto 0.43.1 [34].

Differential gene expression analysis was performed using the R package DESeq2 version 1.32.0 [35]. The estimated number of reads obtained from Kallisto were used as the input for DESeq2. Briefly, DESeq2 normalizes samples according to per-sample sequencing depth and accounting for intrasample variability. Then, it fits data to a negative binomial generalized linear model and calculates the Wald statistic. Finally, the raw *p* values were corrected using the false discovery rate for multiple testing by the Benjamini–Hochberg method. Genes with a false discovery rate of less than 0.05 and an absolute log_2_ fold change of more than one was considered differentially modified. Principal component analysis was performed using the 500 most variable genes, and their expression was normalized using the variance-stabilizing transformation method.

Functional enrichment was evaluated using Fast Gene Set Enrichment Analysis [36], based on the full list of differentially expressed genes that were ranked by the Wald test results, and using the Molecular Signatures Database (MSigDB) as the reference [37]. Among the selected MSigDB gene sets were hallmark and canonical pathways (KEGG and Reactome).

Heatmaps were used to represent the average gene normalized counts in each condition. The gene expression values were scaled using a Z-score normalization (for each condition, the gene expression value was subtracted from the gene average value and divided by its standard deviation [x – mean/standard deviation]).

## Results

### Potency and Selectivity of GLPG3667

Characterization of GLPG3667 at the biochemical level, using different types of assays, indicated selective inhibition of TYK2 and JAK2 over JAK1 and JAK3, with a rank order potency of TYK2 > JAK2 > JAK1 > JAK3 (Table 1). Cellular assays were set up to further characterize the potency and selectivity of GLPG3667 among the JAK family. Table 2 presents the data collected in various cell systems for multiple JAK combinations. First, compound activity was assessed in fresh human PBMCs for JAK1/TYK2-, JAK1/JAK3-, and JAK2-dependent pathways (IFN-α, IL-2, and GM-CSF pathways, respectively) by measuring STAT phosphorylation using flow cytometry. GLPG3667 displayed more than 14- and 13-fold preferential inhibition of the JAK1/TYK2-dependent pathway over the JAK1/JAK3- and JAK2-dependent pathways, respectively, suggesting selectivity for TYK2 inhibition over the three other JAK family members. Using fresh human PBMCs activated for 4 days with PMA and ionomycin, the strong potency of GLPG3667 was confirmed for the TYK2-dependent IL-12 and IL-23 pathways [10], with a potency close to that of the IFN-α pathway. The IL-10 pathway, which depends on JAK1 and TYK2, was shown to be less sensitive to GLPG3667 than other pathways, indicating inefficient inhibition of JAK1. Furthermore, it is noteworthy that GLPG3667 was a weak inhibitor of the IL-22 pathway, a cytokine from the IL-10 family of cytokines that functions via the IL-10 receptor β [38], in a human colorectal adenocarcinoma cell line (HT-29). The IL-4 and IL-13 pathways, which are driven by JAK1 and TYK2 in non-hematopoietic cells [39], also displayed a much lower sensitivity to GLPG3667 in the human lung adenocarcinoma cell line NCI-H1650 than the TYK2-dependent pathways.

**Table 1:** IC_50_ values for the inhibition of recombinant JAK1, JAK2, JAK3, and TYK2 by GLPG3667 were determined by using ADP-Glo Kinase Assays, radioactive filter plate assays, or time-resolved FRET assays, as indicated

Targeted JAK protein	Assay	*N*	Average pIC_50_ ± SEM	IC_50_ (nM)
JAK1	Time-resolved FRET assay	13	7.42 ± 0.05	37.6
JAK2	Time-resolved FRET assay	13	8.14 ± 0.04	7.3
TYK2	Radioactive filter plate assay^a^	7	8.63 ± 0.07	2.3
	ADP-Glo Kinase Assay	7	8.97 ± 0.05	1.1
JAK3	Radioactive filter plate assay^a^	7	6.43 ± 0.06	374
	Radioactive filter plate assay^b^	3	< 5.00	> 10,000

The pIC_50_ is defined as the negative of the log10 of the compound concentration that has a half maximal effect on the readout

Abbreviations: *N* number of individual assays run, *FRET* fluorescence resonance energy transfer, *IC*_*50*_ half maximal inhibitory concentration, *JAK* Janus kinase, *SEM* standard error of the mean, *TYK2* tyrosine kinase 2

^a^Performed at Galapagos

^b^Performed at Eurofins

**Table 2:** IC_50_ values in cellular assays were determined by plotting the compound concentration versus the effect on the pSTAT readouts after triggering the cells with the indicated cytokines for 20–30 min

Cell type	Assay	Targeted JAK protein(s)	*N*	Average pIC_50_ ± SEM	IC_50_ (nM)
Human PBMCs	IFN-α/pSTAT1	JAK1/TYK2	6	7.10 ± 0.05	78.6
	IL-2/pSTAT5	JAK1/JAK3	6	5.95 ± 0.04	1113
	GM-CSF/pSTAT5	JAK2	6	5.99 ± 0.09	1022
Human activated PBMCs	IFN-α/pSTAT1	JAK1/TYK2	3	7.34 ± 0.13	45
	IL-10/pSTAT3	JAK1/TYK2	3	6.51 ± 0.13	311
	IL-12/pSTAT4	JAK2/TYK2	3	6.78 ± 0.02	167
	IL-23/pSTAT3	JAK2/TYK2	3	7.10 ± 0.14	80
HT-29	IL-22/pSTAT1	JAK1/TYK2	3	6.48 ± 0.22	333
	IL-22/pSTAT3	JAK1/TYK2	3	< 5.50	> 3000
NCI-H1650	IL-4/pSTAT6	JAK1/TYK2	12	6.13 ± 0.09	741
	IL-13/pSTAT6	JAK1/TYK2	10	6.36 ± 0.07	432

The pIC_50_ is defined as the negative of the log_10_ of the compound concentration that has a half maximal effect on the readout

Abbreviations: *N* number of individual assays run, *IC*_*50*_ half maximal inhibitory concentration, *GM-CSF* granulocyte-macrophage colony-stimulating factor, *IFN* interferon, *IL* interleukin, *JAK* Janus kinase, *PBMC* peripheral blood mononuclear cell, *pSTAT* phosphorylated signal transducer and activator of transcription, *SEM* standard error of the mean, *TYK2* tyrosine kinase 2

Human whole blood assays were used to further document the potency and selectivity of GLPG3667 in more functional systems. First, the potency of GLPG3667 for inhibiting the release of soluble factors induced by IFN-α, IL-12, and IL-23 was measured. Table 3 shows that the IC_50_ values of GLPG3667 for these pathways were in the same range, demonstrating its capability to inhibit TYK2 with a comparable potency in three different TYK2-dependent pathways in functional assays. Its potency for other pathways in human whole blood assays was evaluated through measurement of STAT phosphorylation after fresh human blood triggering by various cytokines. Data presented in Table 4 demonstrate that GLPG3667 displays a stronger potency for the TYK2-dependent pathway triggered by IFN-α than for pathways dependent on JAK1 (IL-6 pathway), JAK1/JAK3 (IL-2 pathway), JAK2 (GM-CSF pathway), or even JAK1/TYK2 (IL-10 pathway). Globally, GLPG3667 displayed more than 12-fold increased selectivity toward the IL-6 pathway and up to more than 48-fold increased selectivity for the IL-10 pathway when compared with the IFN-α pathway. Altogether, these data establish GLPG3667 as a selective inhibitor of TYK2 over other JAK family members.

**Table 3:** IC_50_ values in human whole blood assays were determined by plotting the compound concentration versus the effect on the IP-10/CXCL10 or IFN-γ released after triggering whole blood with IFN-α, IL-12 + IL-18, and IL-23 + IL-2 + IL-18 for 24 h

Trigger	Assay	Targeted JAK protein(s)	*N*	Average pIC_50_ ± SEM	IC_50_ (nM)
IFN-α	CXCL10	JAK1/TYK2	3	5.9 ± 0.09	1170
IL-12 + IL-18	IFN-γ	JAK2/TYK2	7	6.0 ± 0.05	1000
IL-23 + IL-2 + IL-18	IFN-γ	JAK2/TYK2	4	6.2 ± 0.13	840

The pIC_50_ is defined as the negative of the log_10_ of the compound concentration that has a half maximal effect on the readout

Abbreviations: *CXCL10* C-X-C motif chemokine ligand 10, *N* number of donors assayed, *IC*_*50*_ half maximal inhibitory concentration, *IFN* interferon, *IL* interleukin, *IP-10* interferon-γ-induced protein 10, *JAK* Janus kinase, *SEM* standard error of the mean, *TYK2* tyrosine kinase 2

**Table 4:** IC_50_ values in human whole blood assays were determined by plotting the compound concentration versus the effect on the pSTAT readouts mentioned after triggering the cells with the indicated cytokines for 20–30 min

Cell type	Assay	Targeted JAK protein(s)	*N*	Average pIC_50_ ± SEM	IC_50_ (nM)
CD4^+^	IFN-α/pSTAT1	JAK1/TYK2	13	6.21 ± 0.08	623
CD4^+^	IFN-α/pSTAT3	JAK1/TYK2	3	6.13 ± 0.17	741
CD4^+^	IL-6/pSTAT1	JAK1	6	5.10 ± 0.05	7974
CD4^+^	IL-2/pSTAT5	JAK1/JAK3	3	4.76 ± 0.03	17,512
CD4^+^	IL-10/pSTAT3	JAK1/TYK2	3	< 5.00	> 10,000
CD19^+^	IFN-α/pSTAT3	JAK1/TYK2	3	6.43 ± 0.03	372
CD19^+^	IL-10/pSTAT3	JAK1/TYK2	3	< 5.00	> 10,000
CD33^+^	IFN-α/pSTAT1	JAK1/TYK2	3	6.15 ± 0.09	708
CD33^+^	IL-10/pSTAT3	JAK1/TYK2	3	< 4.5	> 30,000
CD33^+^	GM-CSF/pSTAT5	JAK2	6	4.89 ± 0.05	12,784

The pIC_50_ is defined as the negative of the log_10_ of the compound concentration that has a half maximal effect on the readout

Abbreviations: *GM-CSF* granulocyte-macrophage colony-stimulating factor, *N* number of donors assayed, *IC*_*50*_ half maximal inhibitory concentration, *IFN* interferon, *IL* interleukin, *JAK* Janus kinase, *pSTAT* phosphorylated signal transducer and activator of transcription, *SEM* standard error of the mean, *TYK2 *tyrosine kinase 2

### GLPG3667 Potency in Human in vitro Phenotypic Assays

**Fig. 1:**
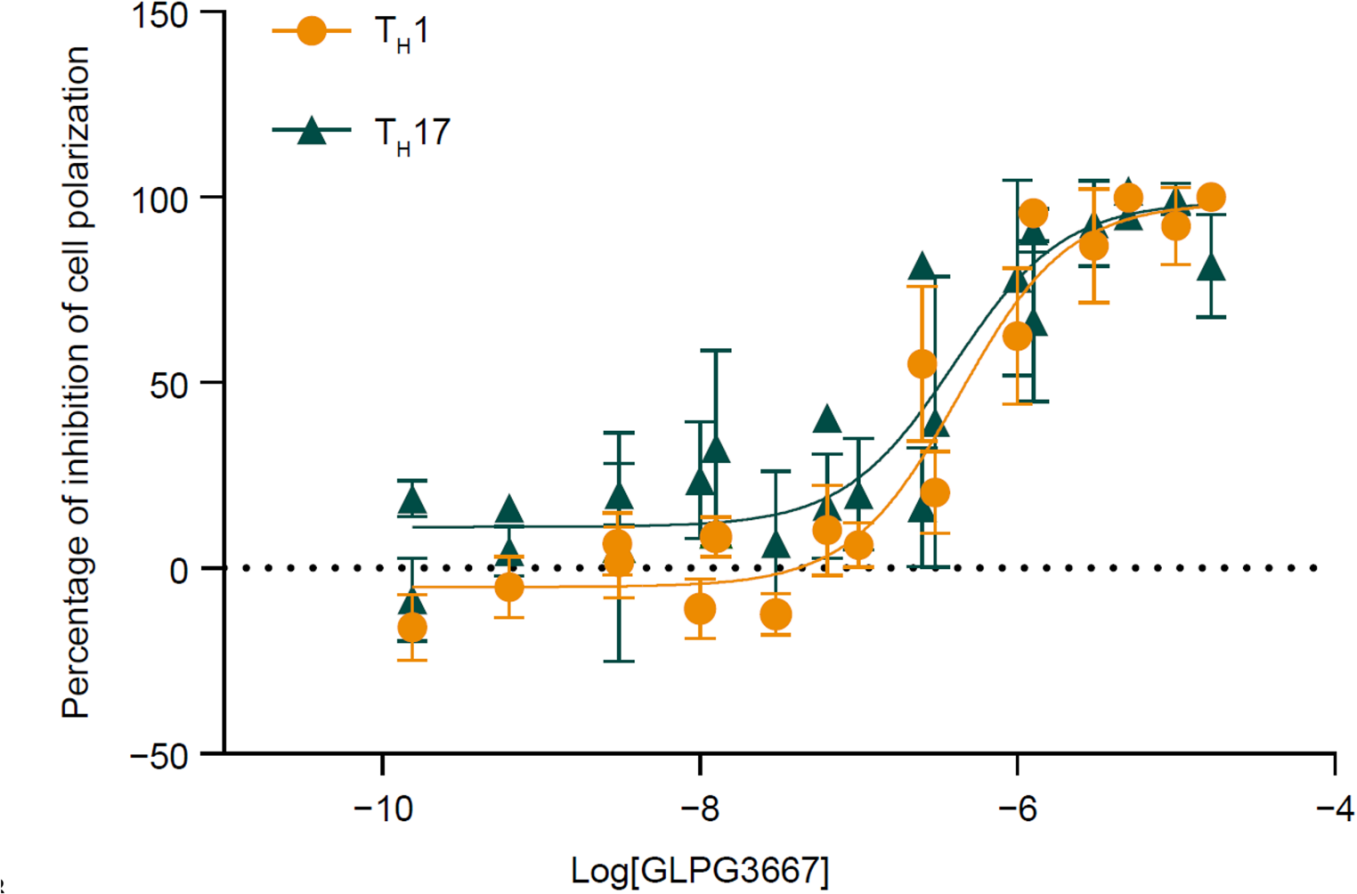
Effect of GLPG3667 on T_H_1 and T_H_17 cell polarization from fresh human CD4^+^ naïve T cells measured by the release of IFN-g and IL-17A release, respectively. Graphs represent the mean ± SEM of 9 and 12 different donors for T_H_1 and T_H_17 cells, respectively. Abbreviations: IFN, interferon; IL, interleukin; SEM, standard error of the mean

IL-12 and IL-23 are required for the polarization of T_H_1 and T_H_17 cells, respectively, from CD4^+^ T cells. Therefore, the capacity of GLPG3667 to have an impact on these processes was analyzed. Naive human CD4^+^ T cells purified from human whole blood were cultured with increasing concentrations of GLPG3667 in the presence of cytokines and antibodies that drive differentiation into T_H_1 or T_H_17 subsets; the release of IFN-γ and IL-17, respectively, was measured in the culture medium. Data presented in Fig. 1 show that GLPG3667 was able to fully inhibit the polarization of both T_H_1 and T_H_17 cells, with IC_50_ values of 398 nM and 224 nM, respectively.

**Fig. 2:**
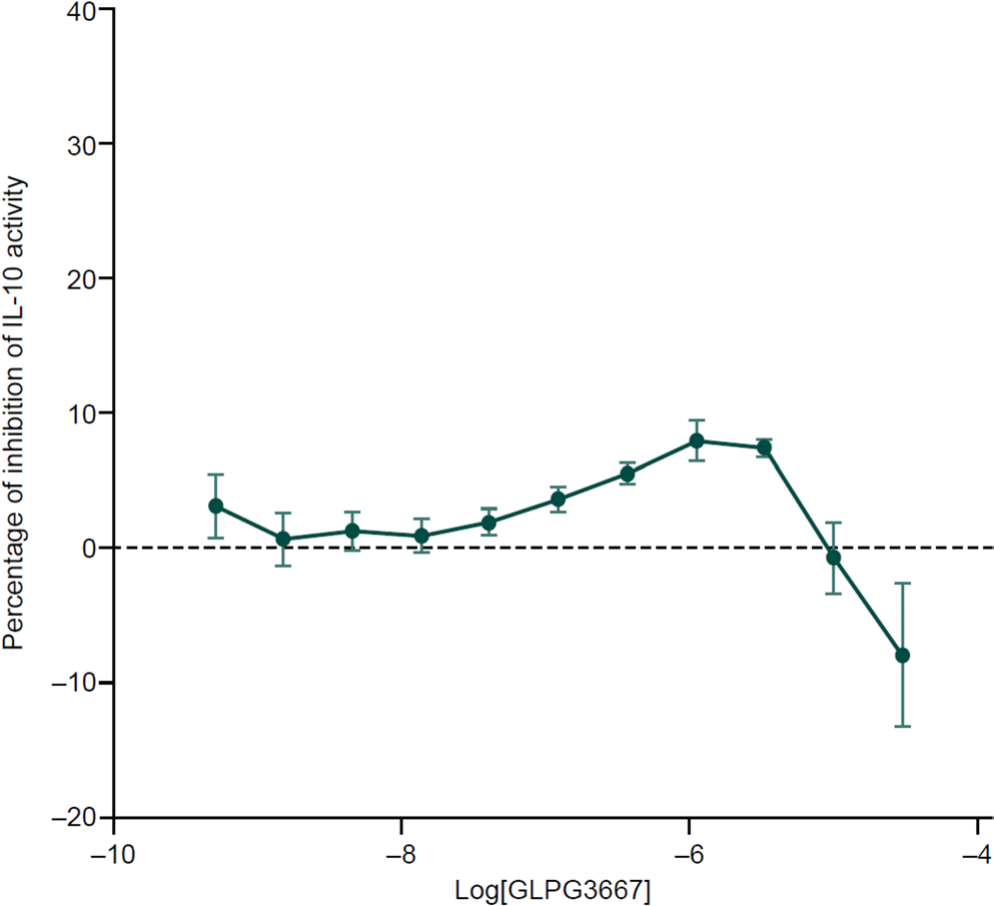
Effect of GLPG3667 on IL-10 phenotypic assay. Lack of inhibition of the IL-10 anti-inflammatory activity of GLPG3667 on LPS-induced TNF-α release in human monocytes. Data are represented as the mean ± SEM of four different donors. Abbreviations: IL, interleukin; LPS, lipopolysaccharide; SEM, standard error of mean; TNF-α, tumor necrosis factor-α

The low potency of GLPG3667 on the IL-10 pathway observed in human PBMCs and whole blood assays prompted us to define if this was also observed in a phenotypic assay designed to analyze the capability of IL-10 to alter TNF-α release from human monocytes triggered with LPS. For this purpose, monocytes purified from human blood were preincubated with increasing doses of GLPG3667 and treated with IL-10, and were finally triggered with LPS for 15 h before measurement of the TNF-α concentration in the culture medium. Figure 2 shows that at doses of up to 10 µM, GLPG3667 failed to have an impact on the blocking effect of IL-10 on LPS-induced TNF-α release. IL-10 dependency was demonstrated by preincubation of IL-10 with neutralizing anti-IL-10 antibody, which recovered more than 90% of the IL-10-induced inhibition of TNF-α release (data not shown). These data confirm that GLPG3667 does not inhibit the anti-inflammatory response of monocytes to IL-10.

### Potency and Selectivity of GLPG3667 after Oral Administration in Healthy Volunteers

To evaluate the impact of GLPG3667 on various JAK-dependent pathways after oral administration in humans, STAT phosphorylation was measured in human whole blood collected from healthy volunteers from the first-in-human phase 1 study (NCT04097938) and a drug–drug interaction phase 1 study (NCT04736927), after ex vivo triggering with various cytokines.

**Fig. 3:**
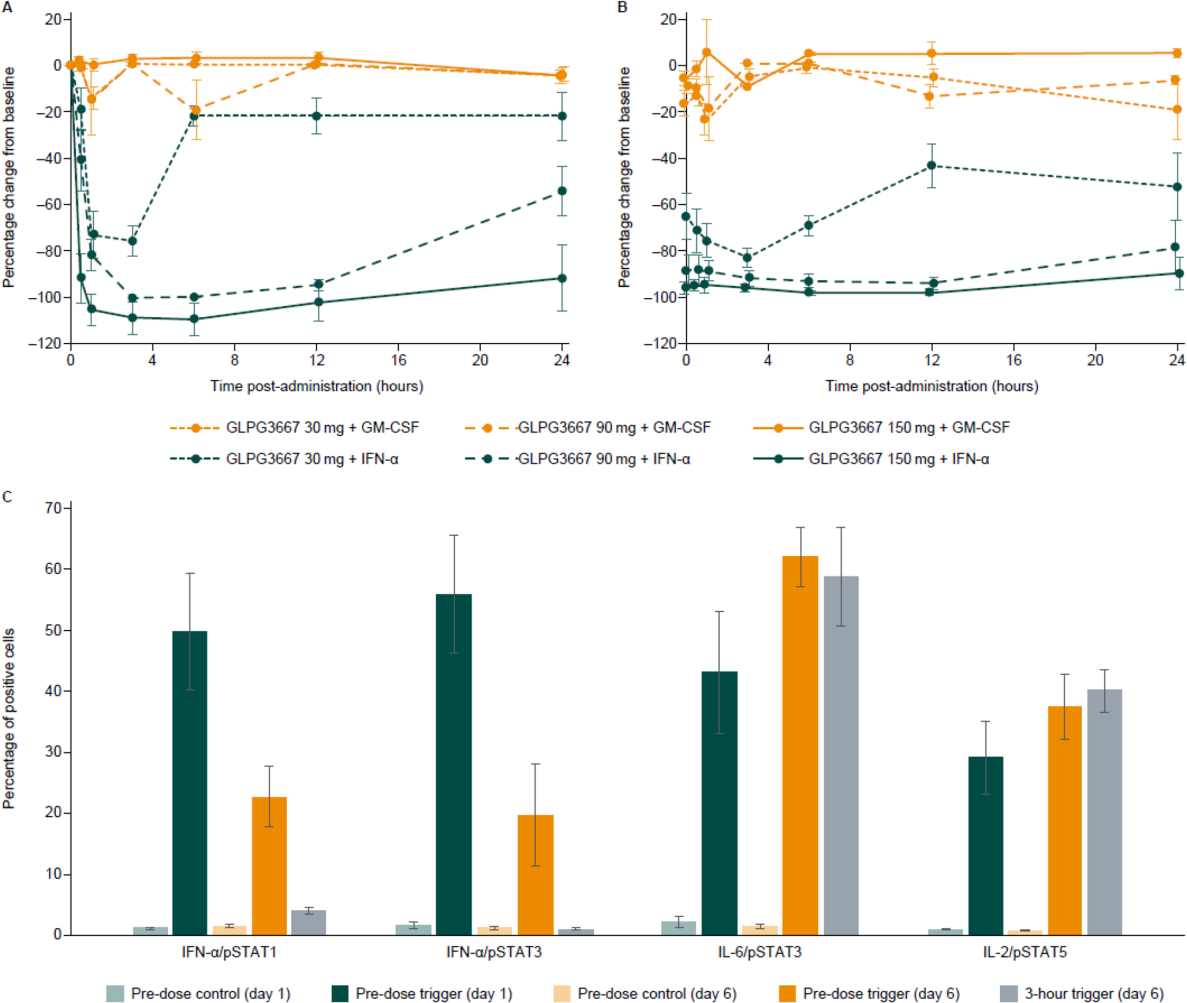
Pharmacodynamic effects of GLPG3667 in healthy human volunteers dosed orally during the first-in-human study (**A** and **B**) and a drug–drug interaction study (**C**). Blood was collected pre-dose and at various time points after compound administration on day 1 (**A**) and day 10 (**B**) and was triggered ex vivo with the indicated cytokines. During the drug–drug interaction study, blood was collected pre-dose on day 1 and day 6, and 3 h post-administration (T_max_) on day 6, and was triggered ex vivo with the indicated cytokines. STAT phosphorylation was measured using flow cytometry and is represented as the mean ± SEM of the percentage of pSTAT-positive cells among CD-positive cells for six donors (**A** and **B**) and 14 donors (**C**). Abbreviations: GM-CSF, granulocyte-macrophage colony-stimulating factor; IFN, interferon; IL, interleukin; pSTAT, phosphorylated signal transducer and activator of transcription; SEM, standard error of the mean

When comparing the impact of a single administration of GLPG3667 in six healthy volunteers at different doses (Fig. 3A), GLPG3667 displayed dose-dependent inhibition of the IFN-α pathway, leading to an almost complete absence of STAT1 phosphorylation over 24 h at the 150 mg dose. In contrast, no effect was observed with any of the three doses on the GM-CSF pathway. After 10 days of administration of GLPG3667 (steady state, Fig. 3B), inhibition of the IFN-α pathway was complete at doses of 90 mg and 150 mg; notably, the inhibition was sustained throughout the day and was still present 24 h after administration of the compound. As with single-dose administration, after 10 days of dosing, no effect was observed on the GM-CSF pathway. These data confirm that GLPG3667 does not alter JAK2-dependent pathways at high doses, whereas the compound is able to fully block the TYK2-dependent IFN-α pathway, when administered orally in healthy volunteers.

The effects of GLPG3667 on various JAK–STAT pathways were then assessed in a second phase 1 study in which the compound was given for 4 days before blood was collected pre-dose and 3 h after the day 4 administration (T_max_ of the molecule in humans). Data presented in Fig. 3C show that, before dosing with GLPG3667 (pre-dose of day 1), IFN-α trigger strongly stimulated phosphorylation of STAT1 and STAT3 compared with the control at the same time point. Similarly, IL-6 and IL-2 strongly induced the phosphorylation of STAT3 and STAT5, respectively. On day 6 (after 4 days of compound administration), IFN-α-induced STAT1 and STAT3 phosphorylation was strongly decreased pre-dose and fully blocked at the T_max_. In contrast, no effect of GLPG3667 administration was observed on IL-6-induced STAT3 phosphorylation and IL-2-induced STAT5 phosphorylation pre-dose and at the T_max_. These data confirm the selectivity of GLPG3667 for TYK2-dependent pathways over JAK1- and JAK1/JAK3-dependent pathways. Altogether, the data establishes that, when given orally at doses that completely block the IFN-α pathway, GLPG3667 does not have an impact on JAK1-, JAK1/JAK3-, and JAK2-dependent pathways and, thus, remains selective for TYK2 over the other JAK family members.

### GLPG3667 Effects in Healthy Volunteers after in vivo IFN-α Challenge

Finally, the capability of orally administered GLPG3667 to alter the biological effects of IFN-α was assessed in healthy volunteers after an in vivo IFN-α challenge. Volunteers received a single subcutaneous injection of IFN-α Intron-A in the abdomen in 30 min after GLPG3667 or placebo administration on day 11. Administration of the compound was continued on days 12 and 13, and blood was collected for serum preparation and gene expression analysis pre-dose on day 11 and at different time points after IFN-α injection (Supplementary Fig. 2).

**Fig. 4:**
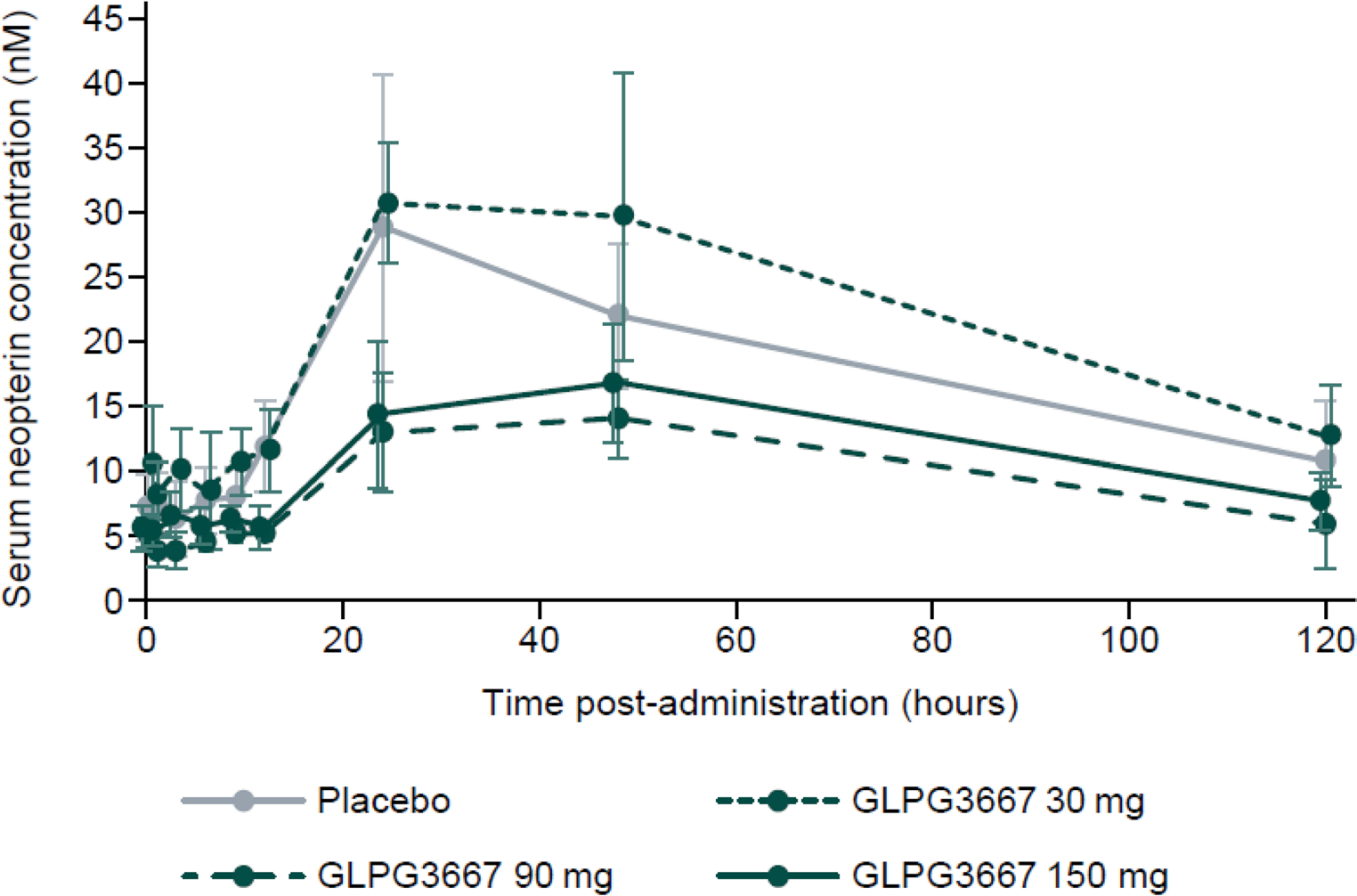
Neopterin concentrations measured in the serum of healthy human volunteers from the first-in-human study following administration of placebo or GLPG3667 and IFN-α challenge. Data are represented as the mean ± standard deviation of six donors receiving placebo or GLPG3667 30 mg, 90 mg, or 150 mg once a day. Abbreviation: IFN, interferon

First, the concentration of neopterin, a chemical that is generated by monocytes in response to IFN-α level increases [40], was measured in the serum of study participants. Figure 4 shows that IFN-α induced generation of neopterin, with a peak at 24 h after injection, and that GLPG3667, when given orally once a day at doses of 90 mg and 150 mg, provided identical inhibition of more than 60% of the IFN-α-induced generation of neopterin. This demonstrates that the maximal effect of GLPG3667 is obtained with a dose of 90 mg of the compound given as a liquid suspension.

**Fig. 5:**
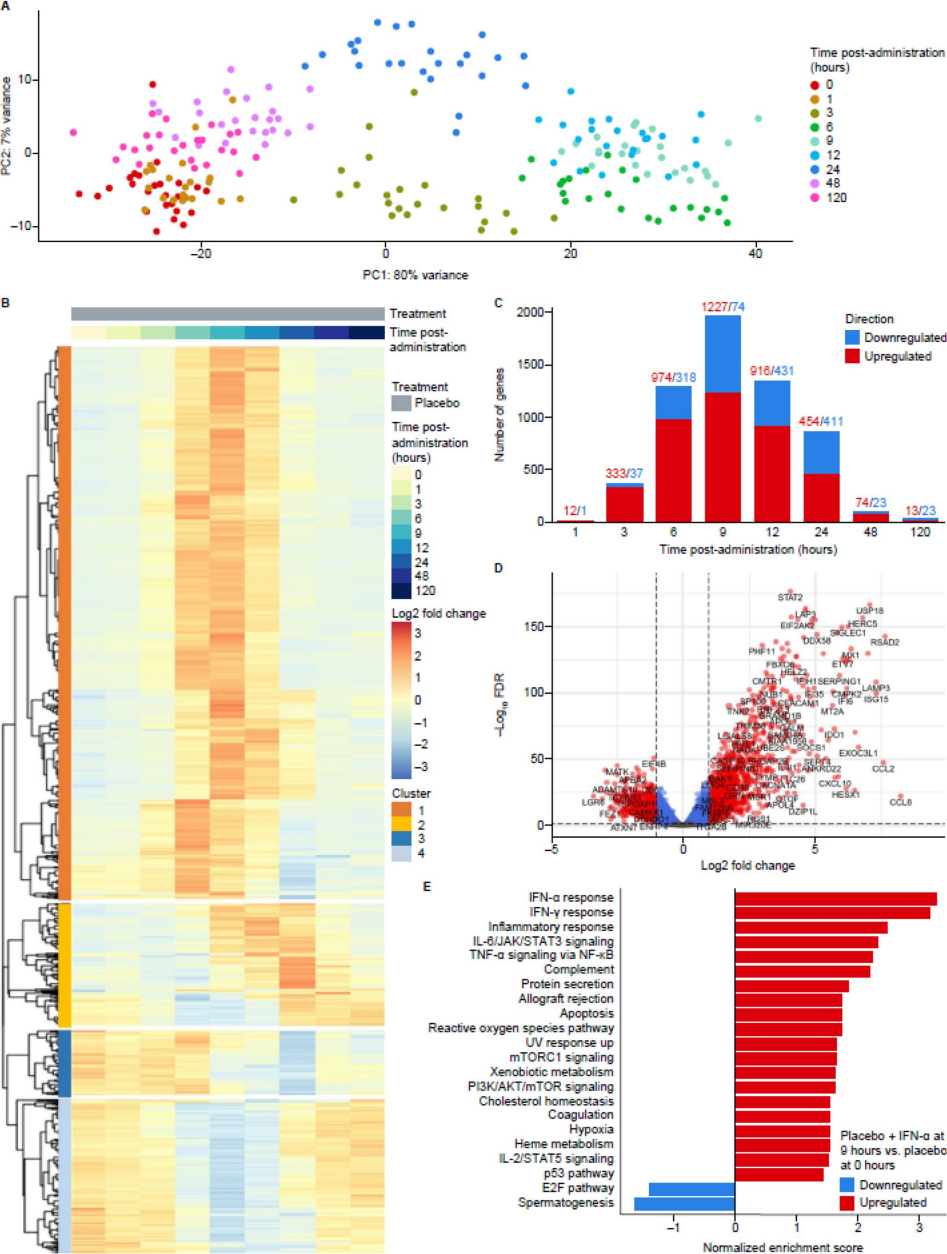
Type I IFN-induced responses evaluated by RNA sequencing in peripheral blood cells of healthy donors. (**A**) PC analysis was performed using the 500 most variable genes. The percentage of variance retained by the two first components is represented. The donor effect was removed (using the function ‘removeBatchEffect’) from the R package limma, before plotting, and the gene expression levels were normalized using the variance-stabilizing transformation. Samples are colored by the time after exposure to IFN-α. Time is represented in hours. (**B**) Heatmap of the top DEGs (log_2_ fold change > 1 and adjusted *p* value < 0.01 compared with baseline) grouped using hierarchical clustering. The gene expression values were scaled using a Z-score normalization (for each condition, the gene expression value was subtracted from the gene average value and divided by its SD [x – mean/SD]). (**C**) Total number of DEGs (log_2_ fold change > 1 and adjusted *p* value < 0.05) induced by IFN-α at each time point. (**D**) Volcano plot of DEGs affected by IFN-α + placebo at 9 h vs. placebo at baseline. Each gene was plotted as log_2_ fold change (x-axis) and −log_10_(adjusted *p* value [FDR]) (y-axis). The horizontal dashed line indicates the cutoff of −log_10_(adjusted *p* value) of > 1.3 (adjusted *p* value < 0.05), and the vertical dashed lines indicate log_2_ fold change > 1. (**E**) Gene set enrichment analysis following differential gene expression analysis of peripheral blood cells exposed to placebo + IFN-α at 9 h vs. placebo at 0 h. Hallmark gene sets upregulated (red) and downregulated (blue) are shown. Only significantly modified pathways (adjusted *p* value < 0.05) are shown as bar plots with the normalized enrichment score on the x-axis. Abbreviations: DEG, differentially expressed gene; FDR, false discovery rate; IFN, interferon; IL, interleukin; JAK, Janus kinase; PC, principal component; SD, standard deviation; STAT, signal transducer and activator of transcription; TNF-α, tumor necrosis factor-α

Next, to evaluate the effects of GLPG3667 in an unbiased way, a genome-wide analysis was performed using RNA sequencing of peripheral blood cells obtained from healthy volunteers receiving placebo or GLPG3667 and who underwent the IFN-α challenge. Initial exploration of the data, through principal component analysis, identified a clear effect of IFN treatment on gene expression over time (Fig. 5A). In agreement with this, differential gene expression analysis highlighted two major clusters of predominantly upregulated genes (clusters 1 and 2; Fig. 5B) and two smaller clusters of downregulated genes (clusters 3 and 4; Fig. 5B). The peak of IFN-α effects among the placebo group was observed at 9 h, with 1974 genes significantly modified (1227 upregulated and 747 downregulated) compared with pre-dose (Fig. 5C, D). Furthermore, functional evaluation using gene set enrichment analysis confirmed that these genes were predominantly involved in the response to IFNs (Fig. 5E).

**Fig. 6:**
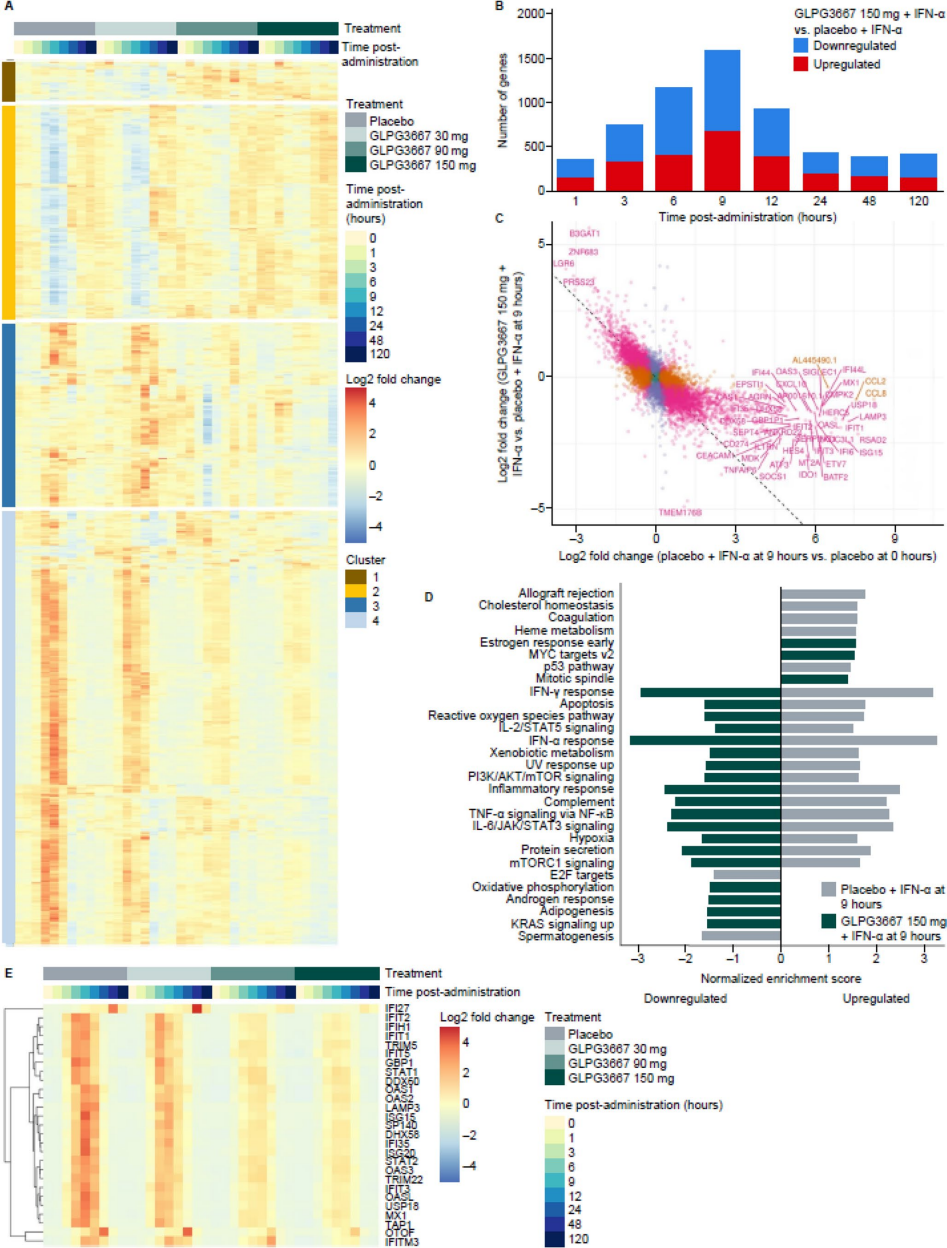
Evaluation of GLPG3667 effects on IFN-α responses in healthy donors by RNA sequencing. (**A**) Heatmap of the top DEGs (log_2_ fold change > 1 and adjusted *p* value < 0.01, GLPG3667 + IFN-α vs. placebo + IFN-α) grouped using hierarchical clustering. The gene expression values were scaled using a Z-score normalization (for each condition, the gene expression value was subtracted from the gene average value and divided by its SD [x – mean/SD]). (**B**) Total number of DEGs (log_2_ fold change > 1 and adjusted *p* value < 0.05) modulated by GLPG3667 (150 mg) + IFN-α vs. placebo + IFN-α at each time point (blue = downregulated, red = upregulated). (**C**) Linear correlation between the log_2_ fold change of GLPG3667 150 mg + IFN-α vs. placebo + IFN-α at 9 h (y-axis) and placebo + IFN-α at 9 h vs. placebo at 0 h (x-axis). Genes that were modified in both contrasts (adjusted *p* value < 0.1) are colored in pink, whereas genes that were only affected by GLPG3667 or placebo are colored in blue and orange, respectively. The dashed line represents a perfect correlation. (**D**) Gene set enrichment analysis following differential gene expression analysis of peripheral blood cells exposed to GLPG3667 150 mg + IFN-α vs. placebo + IFN-α at 9 h (colored in green), and placebo + IFN-α at 9 h vs. placebo at baseline (colored in gray). Hallmark gene sets activated and depleted are shown. Only significantly modified pathways (adjusted *p* value < 0.05) are shown as bar plots with the normalized enrichment score on the x-axis. (**E**) Representation of IFN-responsive genes associated with SLE activity that are affected by GLPG3667 [41]. The gene expression values were scaled using a Z-score normalization (for each condition, the gene expression value was subtracted from the gene average value and divided by its SD [x – mean/SD]). Abbreviations: DEG, differentially expressed gene; IFN, interferon; IL, interleukin; JAK, Janus kinase; SD, standard deviation; SLE, systemic lupus erythematosus; STAT, signal transducer and activation of transcription; TNF-α, tumor necrosis factor-α

After confirming the effects of IFN-α, the impact of GLPG3667 on these responses was evaluated. Interestingly, gene expression analysis in the context of IFN-α exposure showed an opposite pattern with two major clusters of genes inhibited by GLPG3667 (clusters 3 and 4) and two minor clusters in which the downregulation of genes by IFN-α was partially prevented (clusters 1 and 2; Fig. 6A). At the highest dose of GLPG3667 (150 mg), there were 1589 significantly affected genes (927 upregulated and 662 downregulated) after 9 h of treatment (peak of IFN-α effect) in comparison with placebo (Fig. 6B). As suggested in Fig. 6A, these changes generally counteracted the effects observed in the placebo group (Spearman correlation: −0.8) (Fig. 6C). Most importantly, the changes mainly affected genes involved in inflammatory signaling and showed a reversed pattern of pathway activation in comparison with the placebo group (Fig. 6D). In this context, the type I IFN response had the highest inhibitory score among GLPG3667-impacted pathways (Fig. 6D). Furthermore, 25 out of 31 genes associated with SLE activity [41], a disease in which type I IFNs are known to play a key role, were identified as significantly inhibited by GLPG3667 (Fig. 6E). Of note, the RNA sequencing profile also suggested a plateau in the effect starting at GLPG3667 doses of 90 mg (Fig. 6A), which is reinforced by the high similarities in genome-wide changes observed between doses (Spearman correlation: 0.85) (Supplementary Fig. 3). Thus, altogether, these data from phase 1 studies demonstrate that GLPG3667, when given at 90 mg and 150 mg per day, provides strong inhibition of the IFN-α pathway and remains fully selective for TYK2-dependent pathways without affecting other JAK pathways.

## Discussion

JAK1 was initially the preferred therapeutic target compared with other JAK family members owing to its key role in many JAK–STAT pathways. However, JAK1 inhibitors are associated with safety concerns [42]. As a JAK family member involved in few but important pathways that play key roles in inflammation and autoimmunity, TYK2 has since attracted attention, leading to the discovery and development of several inhibitors of this kinase [11, 12, 43]. Indeed, genetic data [13, 17, 19] and approval of antibodies targeting IL-12 and IL-23 (ustekinumab [44]), IL-23 (guselkumab [45]), or type I IFNs (anifrolumab [28]) for the treatment of inflammatory and/or autoimmune diseases generated interest in developing a small molecule that would target these clinically validated pathways. Discovery of GLPG3667 started with the aim of tackling diseases that are dependent on these cytokines, but with the potential to display a better safety profile than previous JAK inhibitors and to provide an orally available alternative to the approved biologic agents.

 Biochemical assay data did not show clear TYK2 selectivity of GLPG3667 over other JAKs, notably over JAK2, whereas cell assays and human whole blood assays did. This is a recurrent observation with biochemical assays, which are generally conducted with purified truncated proteins incubated in synthetic environments with non-physiological ATP concentrations and non-natural substrates [33]. This is indeed also observed with the preferential JAK1 inhibitor filgotinib, which only demonstrated weak selectivity of JAK1 over JAK2 in biochemical assays, however, had minimal JAK2-related adverse events in humans and demonstrated high JAK1 selectivity over JAK2 in human whole blood assays [33]. This also validates the effect of GLPG3667 on TYK2, considering that filgotinib displays weak potency on this kinase. However, data collected in biochemical assays suggested important selectivity toward JAK1 and JAK3 (> 20-fold), which was confirmed in cellular and whole blood assays. Together, our findings demonstrate selectivity of GLPG3667 for TYK2 over other JAK family members. Notably, calculated potencies were higher in biochemical assays than in cell and whole blood assays owing to the absence of serum in the enzymatic reactions. Indeed, a proportion of inhibitor molecules are sequestered by plasma proteins, which decrease the available quantity of inhibitor molecules. The impact of crossing the cell membrane is also absent in these biochemical assays, increasing the capability of the compounds to reach the target.

Interestingly, although GLPG3667 strongly inhibited the JAK1/TYK2-dependent IFN-α pathway in vitro, it moderately or weakly altered other JAK1/TYK2-dependent pathways. Indeed, the IC_50_ values of GLPG3667 for the IL-4, IL-10, IL-13, and IL-22 pathways were 7-fold to 67-fold higher than for the IFN-α pathway. Moreover, the difference in potency for the IL-10 pathway in activated human PBMCs was confirmed in monocytes isolated from human blood and human whole blood, and in a functional assay in human monocytes, in which GLPG3667 was unable to prevent an IL-10-induced decrease of TNF-α release in response to LPS. Notably, the TYK2 inhibitor ropsacitinib was also shown to display a lower potency for the IL-10 pathway than for the IFN-α pathway [11]. These differences were also observed for the IL-13 and IL-22 pathways with deucravacitinib, but not for the IL-10 pathway, in which deucravacitinib displays comparable potency for the IFN-α and IL-10 pathways [12]. In contrast, zasocitinib did not display an effect on the IL-13 and IL-22 pathways in human PBMCs and HT-29 cells, respectively [43]. Finally, using TYK2- and JAK1-selective inhibitors, Sohn et al. showed that the IFN-α, IL-10, and IL-22 pathways were almost strictly dependent on JAK1, with TYK2 inhibitors displaying a very weak potency for these pathways [10]. It is of course very difficult to explain the differences observed with these different TYK2 inhibitors. Most of the data obtained for the IL-22 pathway were collected in HT-29 cells, and this may explain why there is an apparent comparable effect of the TYK2 inhibitors, considering that the cell background is the same or very similar. For the IL-10 pathway, data are available for GLPG3667, ropsacitinib, and deucravacitinib, but are lacking for other TYK2 inhibitors. This prevents defining if the mode of action of these molecules (e.g. allosteric versus non-allosteric inhibition) may be the origin of these differences, notably due to the way of binding to TYK2 and, consequently, changes in protein conformation or the mode of inhibition. However, the similar potency of deucravacitinib for the IFN-α and IL-10 pathways may explain in part its failure in inflammatory bowel disease clinical trials [46], considering that IL-10 is an important modulator of inflammation and epithelial homeostasis in the intestine [47, 48].

The selectivity of GLPG3667 was also assessed in healthy human volunteers, administered orally, during phase 1 studies to verify that it was behaving as expected in various JAK-dependent pathways (Fig. 3). We first confirmed the in vitro data, showing the selectivity for TYK2 over JAK2, and using a second study, which demonstrated that, at the T_max_, GLPG3667 does not alter JAK1- and JAK1/JAK3-dependent pathways. Of interest, at steady state, GLPG3667 was able to completely block the IFN-α pathway even pre-dose, showing that, at doses of 90 mg and 150 mg in suspension formulation, GLPG3667 fully blocked TYK2 activity for 24 h. This means that GLPG3667 can fully block IFN-α activity while not affecting JAK1-, JAK1/JAK3-, and JAK2-dependent pathways when given orally at a dose of 150 mg once a day in a tablet formulation (giving an exposure similar to the 90 mg suspension), thus establishing the TYK2 selectivity of GLPG3667 in humans. It is important to note that evaluation of GLPG3667 activity on different pathways and cell types does not provide a definitive conclusion about its selectivity among the various JAK pathways, even though human whole blood assays are considered the most relevant tests for determining compound potency. However, the absence of effects of GLPG3667 on hematologic parameters observed in a phase 1b study conducted in patients with moderate-to-severe psoriasis (NCT04594928) supports this selectivity of GLPG3667 toward the other JAK family members [49]. Thus, compared with data published by Burke et al. [12] and Leit et al. [43], GLPG3667 displays lower potency and selectivity than deucravacitinib and zasocitinib, respectively, but it seems to be selective enough to avoid other JAK-related effects in humans at the therapeutic doses used in the clinic.

The analysis of human blood samples from the in vivo IFN-α challenge performed during the first-in-human trial first showed that the response to IFN-α in volunteers was comparable to what has already been published. For instance, neopterin is known to be released by monocytes/macrophages in response to type I and type II IFNs and is considered a good biomarker of inflammation [50, 51]. The transcriptomic response to IFN-α also matched the response previously observed and identified as the IFN response genes [41, 52]. Genome-wide comparison of our data with those reported by Rigby et al. [53] identified a significant correlation among the log_2_ changes induced by IFN-α at the time points with higher differentially expressed gene numbers (Spearman correlation: 0.46–0.50), which is an important finding considering the methodological differences among studies (i.e. PBMCs vs. whole blood, in vivo vs. in vitro, and IFN-α dose) (Supplementary Fig. 4). Notably, genes presenting the highest log_2_ fold changes among studies were also the ones most affected by GLPG3667. This in vivo IFN-α challenge confirmed the strong impact of GLPG3667 on type I IFN biological effects. Furthermore, it showed that a plateau is reached with a once-a-day dose of 90 mg given as a suspension, based on neopterin levels in the serum or IFN-α-induced gene expression in blood cells. This further points to the potential of GLPG3667 as a treatment for diseases in which type I IFNs play a key role. The potency and selectivity of GLPG3667 support its evaluation in diseases associated with type I IFN and IL-12/IL-23 dysregulation or activation. The compound displayed strong efficacy in a mouse model of skin inflammation induced by IL-23 associated with a total disappearance of STAT3 phosphorylation in the skin [54]. A short phase 1b study has been conducted with GLPG3667 in patients with moderate-to-severe psoriasis, and data showed efficacy comparable to deucravacitinib and zasocitinib [49, 55, 56]. The pathophysiology of SLE highlights the key role of type I IFNs [57], and the efficacy of anifrolumab [28] and deucravacitinib [24] strongly suggests that blockade of the IFN-α/β pathway through TYK2 inhibition is a valuable way to treat the disease. The neutralizing effect of GLPG3667 on neopterin, which is increased in the serum of patients with SLE and is suspected to be associated with disease severity as well as inhibition of the so-called IFN response signature [58], further supports the study of this compound in patients with SLE. In addition, IL-12 is also considered an important key player in SLE [59], and the role of a polymorphism of STAT4 in patients with SLE [60] further suggests that inhibition of the IL-12 pathway using a TYK2 inhibitor in these patients may be therapeutically advantageous. DM is another disease characterized by an IFN signature [61] that has been shown to correlate with disease severity [62, 63]. It has been recently reported that the anti-IFN-β antibody PF-06823859 provides an acceptable safety profile and efficacy in patients with DM, also pointing out the key role of type I IFNs in this disease [64] and thus the potential effectiveness of a TYK2 inhibitor in patients with this pathology. In addition, increased serum levels of neopterin have been associated with a poor prognosis in patients with DM, further associating disease severity with IFNs [65], and the ability of GLPG3667 to decrease IFN-α-induced expression of neopterin further supports GLPG3667 as a potential treatment for patients with DM. Moreover, the IFN response genes identified in that study and that are downregulated by GLPG3667 are part of the genes constituting the IFN score genes described by Tabata et al. and are independently associated with skin and muscle disease activity in DM [63], which further suggest that GLPG3667 could be a potential treatment for DM.

In conclusion, GLPG3667 is an orthosteric TYK2 inhibitor that demonstrated selectivity and strong potency in orally dosed healthy volunteers, which supports its development for the treatment of patients with DM and SLE.

## Supplementary Information

Below is the link to the electronic supplementary material.

+Supplementary Material 1 (DOCX 467 KB)

## Data Availability

Data obtained from preclinical Galapagos-sponsored research are unavailable to protect intellectual property rights.
