# Supplementary material for: Pharmacological Characterization of GLPG3667, a Tyrosine Kinase 2-Selective Inhibitor, for the Treatment of Inflammatory and Autoimmune Diseases
Source: Inflammation. 2026 May 16;49(1):162. doi: 10.1007/s10753-026-02519-1 (PMC13346301; doi:10.1007/s10753-026-02519-1)
Supplement: Supplementary file 1 — +Supplementary Material 1 (DOCX 467 KB) [file 10753_2026_2519_MOESM1_ESM.docx]

# Supplementary materials

**Pharmacological Characterization of GLPG3667, a Tyrosine
Kinase 2-Selective Inhibitor, for the Treatment of Inflammatory
and Autoimmune Diseases**

**Céline Cottereaux^1,2^** **· May-Linda Lepage^1,2^** **· Isabelle Parent^1,2^** **· Maikel Colli^3^** **· An Van de Water^3^** **· Emilie Lagoutte^1,4^** **· Christelle David^1,5^** **· Maarten Van Balen^3^** **· Adrien Cosson^1,2^** **· Roland Blanque^1,2^** **· Kenji Shoji^1,6^** **· Mia Jans^3^** **· Laetitia Furio^1,4^** **· Steven Van der Plas^3,7^** **· Reginald Brys^3,8^** **· David Amantini^1^** **· René Galien^1^**

^1^ Galapagos SASU, 102 Avenue Gaston Roussel, 93230 Romainville, France

^2^ Present address: NovAliX, Romainville, France

^3^ Galapagos NV, Generaal De Wittelaan L11 A3, 2800 Mechelen, Belgium

^4^ Present address: Calida Therapeutics, Paris, France

^5^ Present address: ISS AG, Integrated Scientific Services, Biel/Bienne, Switzerland

^6^ Present address: Oncodesign Precision Medicine, Dijon, France

^7^ Present address: iTeos Therapeutics, Gosselies, Belgium

^8^ Present address: Agomab Therapeutics, Gent, Belgium

## **Supplementary tables**

## **Supplementary Table 1** Anti-pSTAT and anti-CD antibodies used in cytokine-induced pSTAT measurement in human whole blood

| **Assay** | **Cell type** | **Reagent** | **Provider** |
| --- | --- | --- | --- |
| IL2 | CD4^+^ | APC mouse anti-human CD4 (clone RPA-T4) | BD Pharmingen, San Diego, CA, USA |
|  |  | PE mouse anti-STAT5 (pY694) | BD Pharmingen, San Diego, CA, USA |
| IL-6 | CD4^+^ | APC mouse anti-human CD4 (clone RPA-T4) | BD Pharmingen, San Diego, CA, USA |
|  |  | PE mouse anti-STAT1 (pY701) | BD Pharmingen, San Diego, CA, USA |
| IFN-α | CD4^+^ | APC mouse anti-human CD4 (clone RPA-T4) | BD Pharmingen, San Diego, CA, USA |
|  |  | PE mouse anti-STAT1 (pY701) | BD Pharmingen, San Diego, CA, USA |
|  |  | Alexa Fluor 488 mouse anti-STAT3 (pY705) (clone 4/P-STAT3) | BD Pharmingen, San Diego, CA, USA |
| IFN-α | CD33^+^ | APC mouse anti-CD33 (clone P67.6) | BD Pharmingen, San Diego, CA, USA |
|  |  | PE mouse anti-STAT1 (pY701) | BD Pharmingen, San Diego, CA, USA |
| IFN-α | CD19^+^ | Pacific blue anti-human CD19 (HIB19) | Biolegend, San Diego,  CA, USA |
|  |  | Alexa Fluor 488 mouse anti-STAT3 (pY705) (clone 4/P-STAT3) | BD Pharmingen, San Diego, CA, USA |
| GM-CSF | CD33^+^ | APC mouse anti-CD33 (clone P67.6) | BD Pharmingen, San Diego, CA, USA |
|  |  | PE mouse anti-STAT5 (pY694) | BD Pharmingen, San Diego, CA, USA |
| IL10 | CD33^+^ | APC mouse anti-CD33 (clone P67.6) | BD Pharmingen, San Diego, CA, USA |
|  |  | Alexa Fluor 488 mouse anti-STAT3 (pY705) | BD Pharmingen, San Diego, CA, USA |
|  | CD4^+^ | APC mouse anti-human CD4 (clone RPA-T4) | BD Pharmingen, San Diego, CA, USA |
|  |  | Alexa Fluor 488 mouse anti-STAT3 (pY705) | BD Pharmingen, San Diego, CA, USA |
|  | CD19^+^ | Pacific blue anti-human CD19 (HIB19) | Biolegend, San Diego,  CA, USA |
|  |  | Alexa Fluor 488 mouse anti-STAT3 (pY705) | BD Pharmingen, San Diego, CA, USA |

Abbreviations: APC, allophycocyanin; GM-CSF, granulocyte-macrophage colony-stimulating factor; IFN, interferon; IL, interleukin; PE, phycoerythrin; STAT, signal transducer and activator of transcription

## **Supplementary figures**


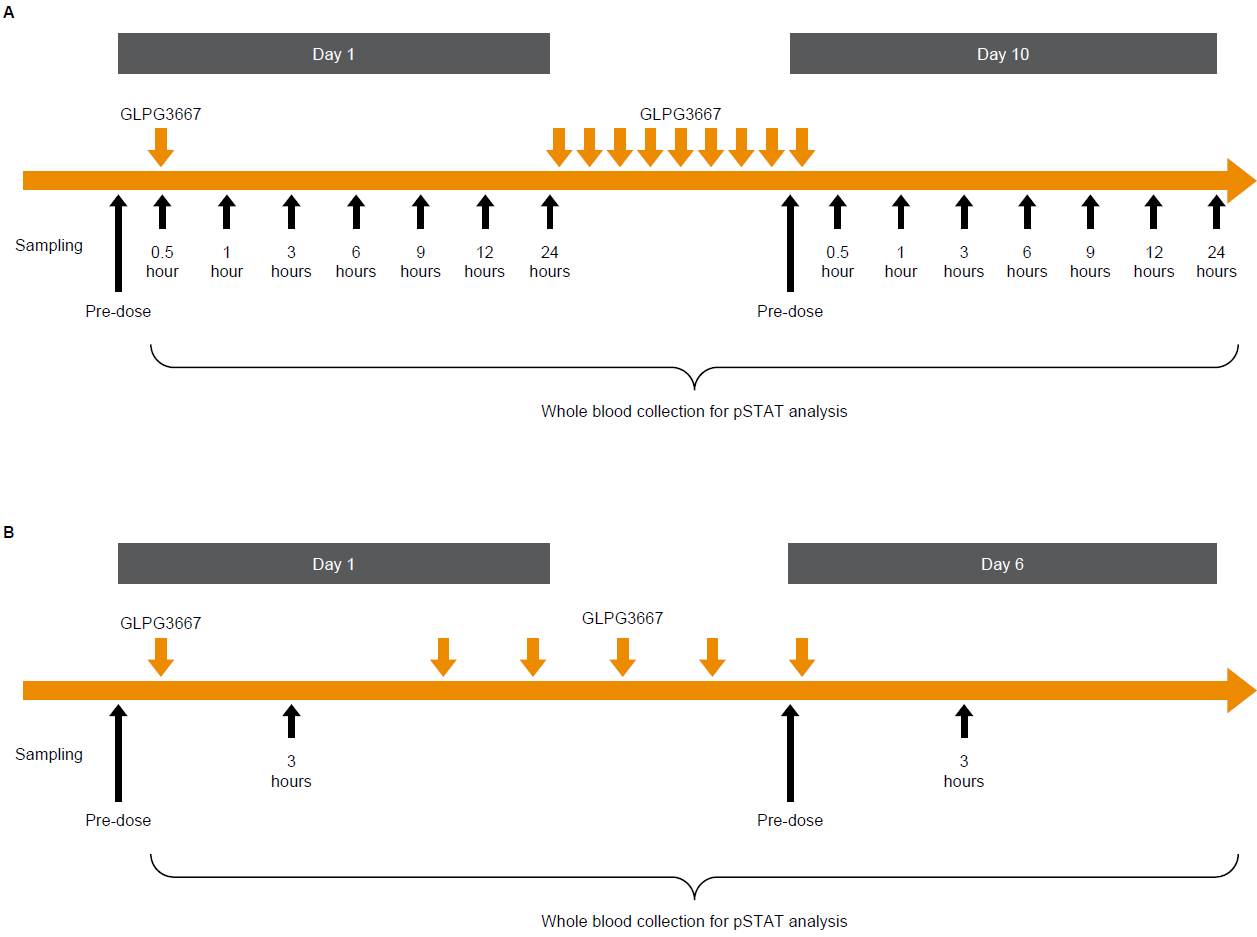


## **Supplementary Fig. 1** Design of the pharmacodynamic analyses performed during the first-in-human study (**A**) and the drug–drug interaction study (**B**), with time points for GLPG3667 administration and blood sampling indicated. Abbreviation: pSTAT, phosphorylated signal transducer and activator of transcription

**
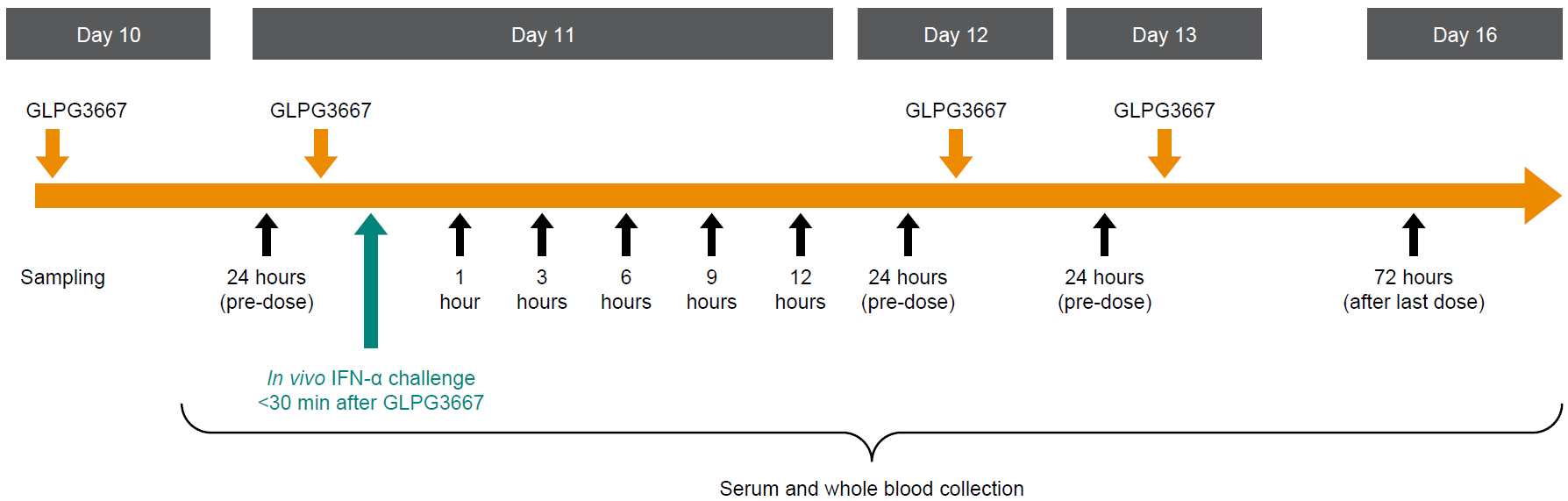
**

## **Supplementary Fig. 2** Design of the in vivo IFN-α challenge performed at the end of the first-in-human study, with time points for GLPG3667 and IFN-α administration and blood sampling indicated. Abbreviation: IFN, interferon

*
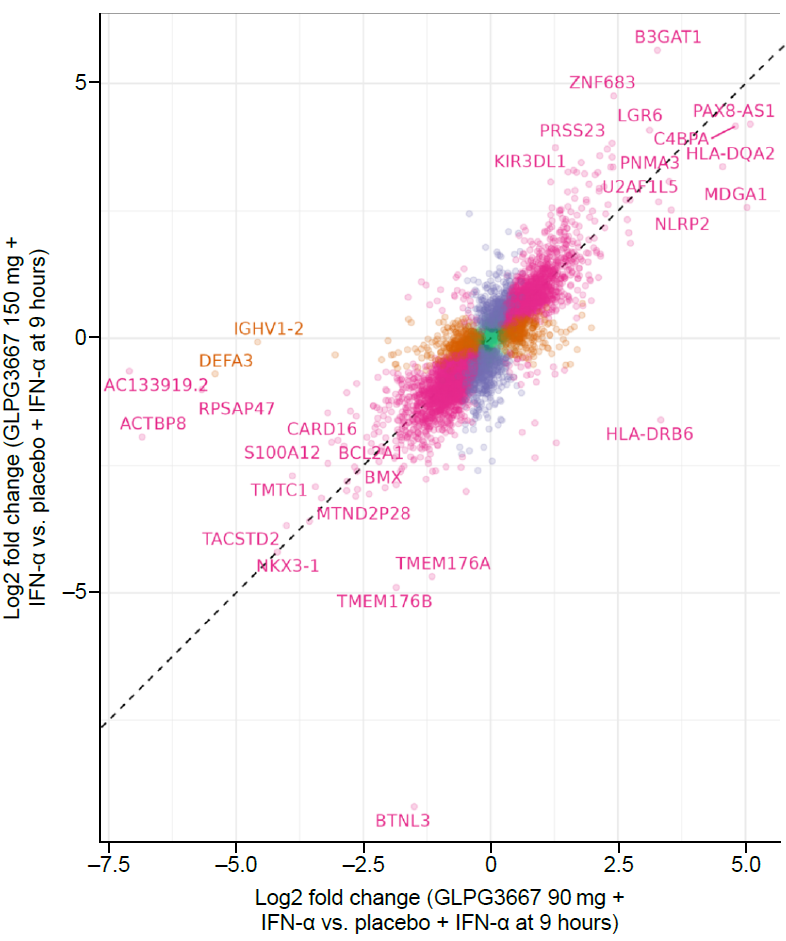
*

## **Supplementary Fig. 3** Comparison of GLPG3667 dose similarities. Linear correlation between the log2 fold change of GLPG3667 150 mg + IFN-α vs. placebo + IFN-α at 9 hours (y-axis) and GLPG3667 90 mg + IFN-α vs. placebo + IFN-α at 9 hours (x-axis). Genes that were modified in both contrasts (adjusted p value < 0.1) are colored in pink, whereas genes that were only affected by GLPG3667 150 mg or 90 mg are colored in blue and orange, respectively. Genes that are not significantly modified in any condition are colored in green. The dashed line represents a perfect correlation. Abbreviation: IFN, interferon


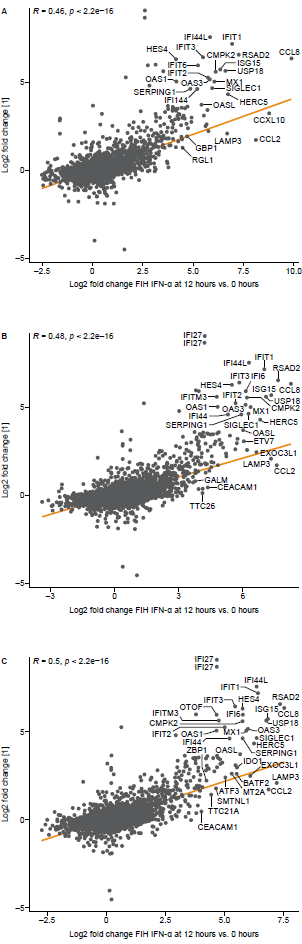


## **Supplementary Fig. 4** Comparison of IFN-α2 effects in the in vivo first-in-human challenge of healthy volunteers and the in vitro exposure of PBMCs (Rigby RE, et al. bioRxiv. 2023; [unpublished results] <https://doi.org/10.1101/2023.07.03.547491>). Spearman correlation of the log2 fold change at the three time points (A: 6 hours, B: 9 hours, and C: 12 hours), presenting the highest number of DEGs after IFN exposure in the current study (x-axis) and the log2 FCs induced in PBMCs exposed or not to IFN-α2 for 24 hours (Rigby et al.) (y-axis). Top upregulated genes are highlighted (log2 fold change > 4 in at least one condition). Abbreviations: DEG, differentially expressed gene; FIH, first-in-human; IFN, interferon; PBMC, peripheral blood mononuclear cell
